# Optimization and clinical validation of a pathogen detection microarray

**DOI:** 10.1186/gb-2007-8-5-r93

**Published:** 2007-05-28

**Authors:** Christopher W Wong, Charlie Lee Wah Heng, Leong Wan Yee, Shirlena WL Soh, Cissy B Kartasasmita, Eric AF Simoes, Martin L Hibberd, Wing-Kin Sung, Lance D Miller

**Affiliations:** 1 Genomic Technologies, Genome Institute of Singapore, Republic of Singapore; 2 Computational and Mathematical Biology, Genome Institute of Singapore, Republic of Singapore; 3 Infectious Diseases, Genome Institute of Singapore, Republic of Singapore; 4 Hasan Sadikin Hospital, Department of Pediatrics, Faculty of Medicine Universitas Padjadjaran, Indonesia; 5 Section of Infectious Diseases, The University of Colorado at Denver and Health Sciences Center and The Children's Hospital, Denver, CO 80262, USA

## Abstract

DNA microarrays used as 'genomic sensors' have great potential in clinical diagnostics. Biases inherent in random PCR-amplification, cross-hybridization effects, and inadequate microarray analysis, however, limit detection sensitivity and specificity. Here, we have studied the relationships between viral amplification efficiency, hybridization signal, and target-probe annealing specificity using a customized microarray platform. Novel features of this platform include the development of a robust algorithm that accurately predicts PCR bias during DNA amplification and can be used to improve PCR primer design, as well as a powerful statistical concept for inferring pathogen identity from probe recognition signatures. Compared to real-time PCR, the microarray platform identified pathogens with 94% accuracy (76% sensitivity and 100% specificity) in a panel of 36 patient specimens. Our findings show that microarrays can be used for the robust and accurate diagnosis of pathogens, and further substantiate the use of microarray technology in clinical diagnostics.

## Abstract 2

New design and optimization of pathogen detection microarrays is shown to allow robust and accurate detection of a range of pathogens. The customized microarray platform includes a method for reducing PCR bias during DNA amplification.

## Background

Timely, accurate and sensitive detection of infectious disease agents is still difficult today, despite a long history of progress in this area. Traditional methods of culture and antibody-based detection still play a central role in microbiological laboratories despite the problems of the delay between disease presentation and diagnosis, the limited number of organisms that can be detected by these approaches, and the 'hit-or-miss' nature of the diagnostic process, which depends on a clinical prediction of the infectious source [1]. Faster diagnosis of infections would reduce morbidity and mortality, for example, through the earlier implementation of appropriate antimicrobial treatment. During the past few decades, various methods have been proposed to achieve this, with those based on nucleic acid detection, including PCR and microarray-based techniques, seeming the most promising. These approaches are beginning to rapidly decrease laboratory turnaround times so that results can be available within 2-6 hours compared to perhaps 24 hours. Future developments may see this reduced even further; and through the development of point-of-care devices, perhaps enable the clinician to make the diagnosis directly at the bed-side [2,3].

While pathogen microarrays and their utility in discovering emerging infectious diseases such as SARS have been described, technical problems related to accuracy and sensitivity of the assay prevent their routine use in patient care [4-9]. For microarrays to become a standard diagnostic tool, the following questions must be addressed: what are the factors that influence probe design and performance? How is a pathogen 'signature' measured and detected? What is the specificity and sensitivity of an optimized detection platform? Can detection algorithms distinguish co-infecting pathogens and closely related viral strains? [10-12].

Noisy signals caused by cross-hybridization artifacts present a major obstacle to the interpretation of microarray data, particularly for the identification of rare pathogen sequences present in a complex mixture of nucleic acids. For example, in clinical specimens, contaminating nucleic acid sequences, such as those derived from the host tissue, will cross-hybridize with pathogen-specific microarray probes above some threshold of sequence complementarity. This can result in false-positive signals that lead to erroneous conclusions. Similarly, the pathogen sequence, in addition to binding its specific probes, may cross-hybridize with other non-target probes (that is, probes designed to detect other pathogens). This latter phenomenon, though seemingly problematic, could provide useful information for pathogen identification to the extent that such cross-hybridization can be accurately predicted. With various metrics to assess annealing potential and sequence specificity, microarray probes have traditionally been designed to ensure maximal specific hybridization (to a known target) with minimal cross-hybridization (to non-specific sequences). However, in practice we have found that many probes, though designed using optimal *in silico *parameters, do not perform according to expectations for reasons that are unclear (CW Wong *et al*., unpublished data).

Here, we report the results of a systematic investigation of the complex relationships between viral amplification efficiency, hybridization signal output, target-probe annealing specificity, and reproducibility of pathogen detection using a custom designed microarray platform. Our findings form the basis of a novel methodology for the *in silico *prediction of pathogen 'signatures', shed light on the factors governing viral amplification efficiency and demonstrate the important connection between a viral amplification efficiency score (AES) and optimal probe selection. Finally, we describe a new statistics-based pathogen detection algorithm (PDA) to link this all together, permitting confident identification of organisms entirely by prediction, and evaluate the entire platform in relation to conventional PCR techniques in a cohort of patients with lower respiratory illness.

## Results and discussion

### Empirical determination of cross-hybridization thresholds on a pathogen detection microarray

To systematically investigate the dynamics of array-based pathogen detection, we created an oligonucleotide array using Nimblegen array synthesis technology [13]. The array was designed to detect up to 35 RNA viruses using 40-mer probes tiled at an average 8-base resolution across the full length of each genome (53,555 probes; Figure S1 and Table S1 in Additional data file 1). Together with 7 replicates for each viral probe, and control sequences for array synthesis and hybridization (see Materials and methods), the array contained a total of 390,482 probes. Initially, we studied virus samples purified from cell lines, reverse-transcribed and PCR-amplified with virus-specific primers (instead of random primers). This allowed us to study array hybridization dynamics in a controlled fashion, without the complexity of cross-hybridization from human RNA and random annealing dynamics, which occur with random primers. We then applied our findings to clinical samples amplified using random primers.

**Figure 1:**
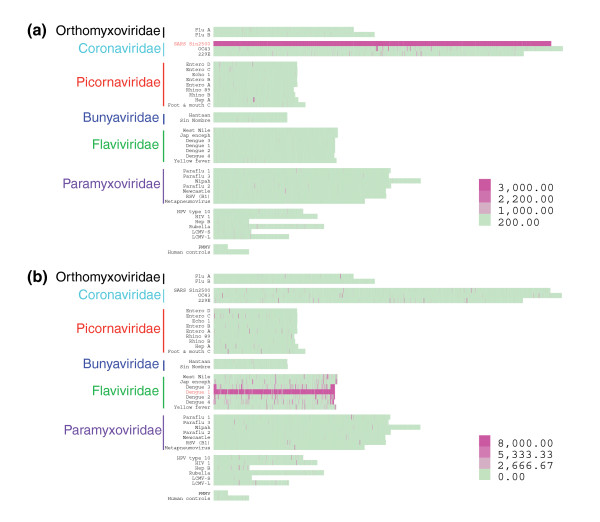
Heatmap of microarray probe signal intensities. Cells corresponding to probes are aligned in genomic order and colored according to the signal intensity-color scales shown. Hybridization signatures corresponding to **(a) **SARS Sin850 or **(b) **Dengue 1 Hawaiian isolate are shown.

SARS coronavirus and Dengue serotype 1 genomic cDNA were amplified in entirety (as confirmed by sequencing), labeled with Cy3 and hybridized separately on microarrays. The SARS sample hybridized well to the SARS tiling probes, with all 3,805 SARS-specific probes displaying fluorescent (Cy3) signal well above the detection threshold (determined by probe signal intensities >2 standard deviations (SD) above the mean array signal intensity; Figure 1a). Cross-hybridization with other pathogen probe sets was minimal, observed only for other members of Coronaviridae and a few species of Picornaviridae and Paramyxoviridae, consistent with the observation that SARS shares little sequence homology with other known viruses [14]. The hybridization pattern of Dengue 1, on the other hand, was more complex (Figure 1b). First, we observed that hybridization to the Dengue 1 probe set was partially incomplete (that is, there were regions absent of signal) due to sequence polymorphisms. The Dengue 1 sample hybridized on the array was cultured from a 1944 Hawaiian isolate, whereas the array probe set was based on the sequence of a Singaporean strain S275/90, isolated in 1990 [15]. Sequencing the entire genomes of these 2 isolates revealed that the array probes that failed to hybridize each contained at least 3 mismatches (within a 15-base stretch) to the sample sequence. Second, we observed that cross-hybridization occurred to some degree with almost all viral probe sets present on the array, particularly with probes of other Flaviviridae members, consistent with the fact that the 4 Dengue serotypes share 60-70% homology. To understand the relationship between hybridization signal output and annealing specificity, we first compared all probe sequences to each viral genome using two measures of similarity: probe hamming distance (HD) and maximum contiguous match (MCM). HD measures the overall similarity distance of two sequences, with low scores for similar sequences [16,17]. MCM measures the number of consecutive bases that are exact matches, with high scores for similar sequences [17,18].

**Figure 2:**
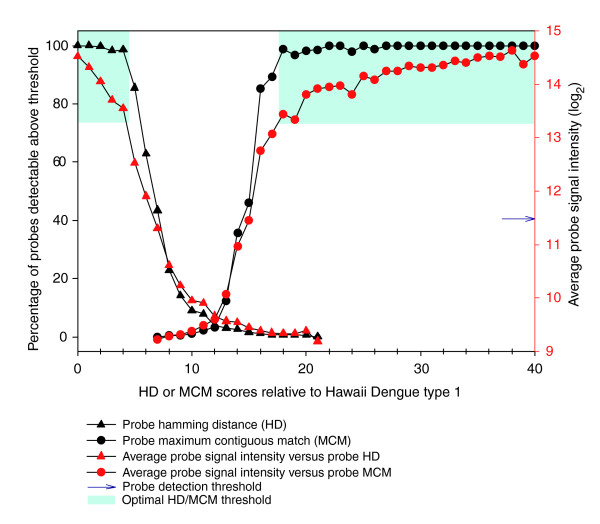
Relationship between probe HD, probe MCM and probe signal intensity. Average probe signal intensity and percentage of detectable probes (signal intensity > mean + 2 SD) decreases as HD increases and MCM decreases. The optimal cross-hybridization thresholds HD ≤ 4 or MCM ≥ 18, where >98% of probes can be detected, is shaded in blue.

We calculated the HD and MCM scores for every probe relative to the Hawaiian Dengue 1 isolate and observed that these scores correlated negatively (HD) and positively (MCM) with probe signal intensity (Figure 2). All probes on the array with high similarity to the Hawaiian Dengue I genome, that is, HD ≤ 2 (*n *= 942) or MCM ≥ 27 (*n *= 627), hybridized with median signal intensity 3 SD above detection threshold. Although 98% of probes were detectable at the low HD range from 0-4, or high MCM range from 18-40, median probe signal intensity decreased at every increment of sequence distance (Figure 2). Median signal intensity dropped off sharply to background levels at HD = 7 and MCM = 15, with 43% and 46% detectable probes, respectively. The majority of probes (>96%, n > 51,000) had HD scores between 8 and 21 and/or MCM scores between 0 and 15, of which only 1.23% and 1.57%, respectively, were detectable.

At the optimal similarity thresholds HD ≤ 4 and MCM ≥ 18, >98% of probes could be detected with median signal intensity 2 SD above detection threshold, whereas adjusting the similarity threshold down 1 step to HD ≤ 5 and MCM ≥ 17 would result in only approximately 85% probe detection and median signal intensity approximately 1.2 SD above detection threshold (Figure 2). Using these optimal HD and MCM thresholds to guard against cross-hybridization, we binned all probes into specific 'recognition signature probe sets' (that is, r-signatures) most likely to specifically detect a given pathogen, and we defined r-signatures for each of the 35 pathogen genomes represented on the array (Table 1). Each pathogen's r-signature comprised tiling probes derived from its genome sequence (HD = 0, MCM = 40), as well as cross-hybridizing probes derived from other pathogens (HD ≤ 4, MCM ≥ 18). According to these criteria, a given probe could belong to multiple different r-signatures, thereby maximizing probe-level evidence for pathogen detection.

**Table 1:** Binning of probes into specific pathogen signature probe sets

	Pathogen	Family	Genome size (nt)	Total tiling probes	Top 20% AES* (a)	GC content filter (b)	Human genome filter (c)	No. of filtered probes left (d = a - (b + c))	No. of predicted cross-hybridizing probes (HD ≤ 4 and MCM ≥ 18) (e)	No. of probes in pathogen r-signature (d + e)
1	LCMV	Arenaviridae	10,056	1,283	348	2	8	338	0	338
2	Hantaan	Bunyaviridae	6,533	834	156	5	5	146	6	152
3	Sin Nombre	Bunyaviridae	6,562	837	182	1	2	179	6	185
4	229E	Coronaviridae	27,317	3,495	494	11	11	472	0	472
5	OC43	Coronaviridae	30,738	3,937	634	15	22	597	3	600
6	SARS	Coronaviridae	29,711	3,805	575	8	2	565	1	566
7	Dengue serotype 1	Flaviviridae	10,717	1,370	230	2	8	220	8	228
8	Dengue serotype 2	Flaviviridae	10,722	1,370	241	0	9	232	11	243
9	Dengue serotype 3	Flaviviridae	10,707	1,370	230	0	4	226	13	239
10	Dengue serotype 4	Flaviviridae	10,649	1,361	229	1	7	221	3	224
11	Japanese encephalitis	Flaviviridae	10,976	1,404	310	3	2	305	12	317
12	West Nile	Flaviviridae	10,962	1,401	320	2	2	316	9	325
13	Yellow fever	Flaviviridae	10,862	1,389	255	2	3	250	2	252
14	Hepatitis B	Hepadnaviridae	3,215	409	147	14	0	133	0	133
15	Influenza A^†^	Orthomyxoviridae	12,561	1,582	510	1	15	494	0	494
16	Influenza B	Orthomyxoviridae	14,452	1,822	665	5	18	642	2	644
17	Human papillomavirus type 10	Papillomaviridae	7,919	1,011	287	16	9	262	0	262
18	hMPV	Paramyxoviridae	13,335	1,705	322	44	17	261	0	261
19	Newcastle disease	Paramyxoviridae	15,186	1,943	329	0	2	327	3	330
20	Nipah	Paramyxoviridae	18,246	2,335	389	12	5	372	0	372
21	Parainfluenza 1	Paramyxoviridae	15,600	1,995	330	8	13	309	2	311
22	Parainfluenza 2	Paramyxoviridae	15,646	2,002	333	10	2	321	0	321
23	Parainfluenza 3	Paramyxoviridae	15,462	1,979	409	28	23	358	3	361
24	RSV B	Paramyxoviridae	15,225	1,948	383	28	4	351	4	355
25	Echovirus 1	Picornaviridae	7,397	945	238	1	10	227	22	249
26	Enterovirus A	Picornaviridae	7,413	946	193	3	0	190	8	198
27	Enterovirus B	Picornaviridae	7,389	944	179	0	4	175	22	197
28	Enterovirus C	Picornaviridae	7,401	945	183	0	0	183	4	187
29	Enterovirus D	Picornaviridae	7,390	944	155	0	3	152	8	160
30	Foot and mouth disease	Picornaviridae	8,115	1,036	194	14	3	177	0	177
31	Hepatitis A	Picornaviridae	7,478	955	163	1	6	156	0	156
32	Rhinovirus A (type 89)	Picornaviridae	7,152	913	191	6	6	179	1	180
33	Rhinovirus B	Picornaviridae	7,212	920	197	2	2	193	0	193
34	HIV 1	Retroviridae	9,181	1,174	191	4	0	187	0	187
35	Rubella	Togaviridae	9,755	1,246	117	65	0	52	0	52
Total			419,242	53,555				9768		9921

*AES scores for all tiling probes were ranked together and only those probes in the top 20th percentile were retained. ^†^Segment 7 of Influenza A was omitted during probe design.

We next considered other non-specific hybridization phenomena that could affect performance of our r-signature probes. For example, we observed a linear relationship between probe signal and %GC content (data not shown). Consistent with previous observations, we found that probes <40% GC hybridized with diminished signal intensities, while probes with >60% GC content showed higher signal intensities [19,20]. Thus, we censored probes with GC <40% or >60% from the r-signatures, despite optimal HD or MCM values. Furthermore, as cross-hybridization with human sequences could also confound results, we compared all probes to the human genome assembly (build 17) by BLAST using a word size of 15 [21]. Probes with an expectation value of 100 were also censored (Table 1).

While the ideal pathogen r-signature would be one where all probes would hybridize to the target sequence at detectable levels, polymorphic variation between the probes (derived from a consensus sequence) and the actual target would be expected to impede the performance of the r-signature probes at some level. To test this hypothesis, we compared the ratios of detectable to undetectable probes across all r-signatures in the context of the hybridization involving the Hawaiian Dengue 1 isolate. Although the Dengue 1 sequence used to derive the Dengue 1 r-signature was approximately 5% different from the Hawaiian isolate, the detectable probe ratio of the Dengue 1 specific probes was 151/152 (99%), 12 times higher then that for the nearest Dengue serotype signature, suggesting that moderate polymorphic variation is quite tolerable, allowing, in this case, for discernment of the correct pathogen.

### Predicting genome-wide amplification bias

**Figure 3:**
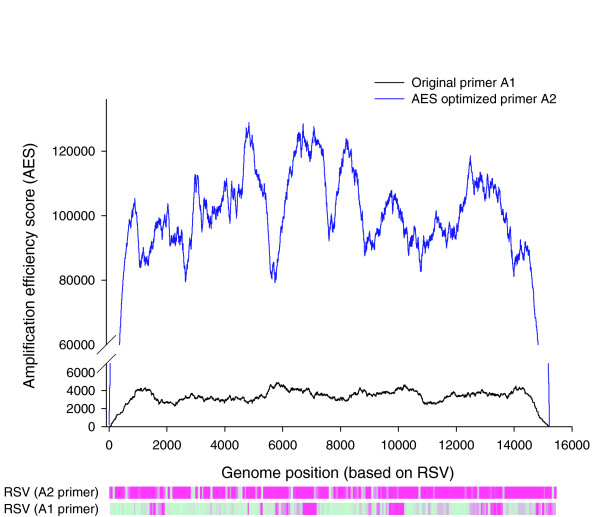
Measurement and application of AES. An RSV patient sample was amplified using original primer A1 (black line), or AES-optimized primer (blue line). The probes that have detectable signal above threshold are shown in purple in the corresponding heatmaps. For primer A1, the detectable regions correspond to regions that have higher AES scores than undetectable regions.

Random priming amplification, rather than primer-specific amplification, is preferred for identifying unknown pathogens in clinical specimens. However, in initial experiments using random priming amplification to identify known pathogens, we frequently observed incomplete hybridization of the pathogen genome marked by interspersed genomic regions not detected by the probes. An example involving the amplification of respiratory syncytial virus (RSV) B from a human nasopharyngeal aspirate is shown in Figure 3. In preliminary analyses, sequence polymorphisms, probe GC content and genome secondary structure failed to explain this phenomenon, suggesting that it might result from a PCR-based amplification bias stemming from differential abilities of the random primers to bind to the viral genome at the reverse transcription (RT) step. The random primer used in our experiments was a 26-mer composed of a random nonamer (3') tagged with a fixed 17-mer sequence (5'-GTTTCCCAGTCACGATA) [4,9,22]. Intra-primer secondary structure formation, such as dimer and hairpin formation between the 17-mer tag and nonamer, and probe melting temperature are known to influence binding efficiency [23,24]. To explore our hypothesis, we designed an algorithm to model the RT-PCR process using experimental data (see Additional data file 1 for details). Briefly, it calculates the probability that a 500-1,000 base-pair product (average size range of PCR product) can be generated from each possible starting position in the genome assuming that a nonamer in the random primer mix will complement the viral sequence perfectly. This probability is reduced when intra-primer hairpin formation is predicted, and increased according to degree of complementarity between tag sequence and viral sequence. In this manner, the probability that each nucleotide will be successfully PCR-amplified is reflected in its AES (see supplemental methods in Additional data file 1 and [25]). To validate the algorithm, we ranked the hybridization signal intensities for all 1,948 probes tiled across the RSV B genome and compared them to their AES values (Figure 3). We observed that high AES significantly correlates to probe hybridization signal intensity above the detection threshold (*P *= 2.2 × 10^-16^; Fisher's exact test). In another experiment involving a patient sample positive for metapneumovirus (hMPV), the probes tiled across the hMPV genome showed a similar result, *P *= 1.3 × 10^-9^. Repeatedly, we observed that higher AES correlated with greater probe detection, with, on average, >70% detection for probes in the top 20% AES (see supplemental methods in Additional data file 1).

**Figure 4:**
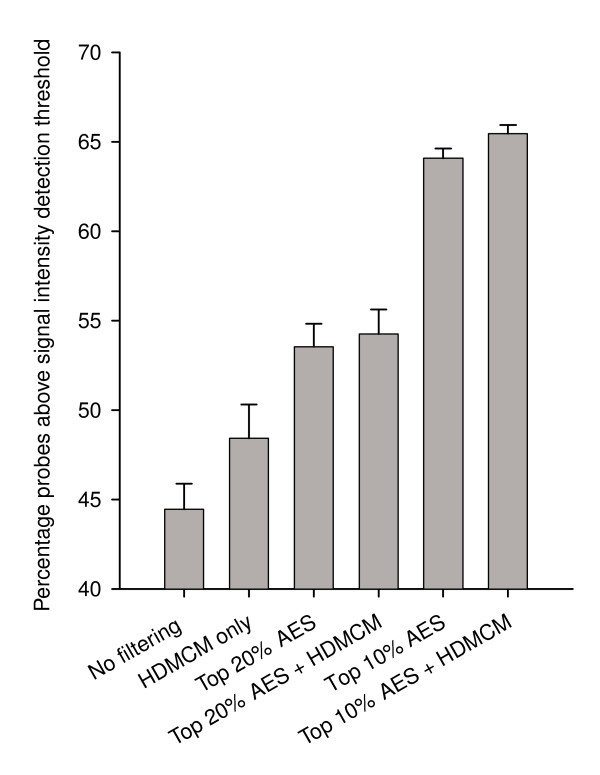
Effects of probe filtering criteria on r-signature probe detection. The 1,948 probes tiled across the RSV B genome were binned according to different filtering criteria and plotted against the percentage of probes with detectable signal. Measurements reflect the average of five experiments.

While HD, MCM, %GC and sequence uniqueness were valuable parameters for probe selection, they did not take into account PCR bias, and were insufficient predictors of probe performance when considered in the absence of AES (Figure 4). We found that using only the probes within the top 20% AES (Table 1) substantially improved the efficacy of our prediction algorithm (discussed in the following section). In total, after applying all probe selection criteria, the r-signatures utilized 9,768 of the >50,000 unique probes initially included on the array.

We next hypothesized that amplification efficiency scoring could be used to select an optimal tag sequence (that is, for the RT-PCR primers) for achieving uniformly high AES across viral genomes, thus globally maximizing PCR efficiency (see supplemental methods in Additional data file 1 and [25]). Briefly, we generated 10,000 primer sequences, eliminated those that formed self-dimers, and calculated AES for every genome based on each candidate primer tag. Primer A2, which had the highest average AES for all 35 viruses present on the array, was selected as the 'AES-optimized' primer. In a comparative study of eight patient samples (five RSV, three hMPV), we observed that primer A2 showed a marked improvement in overall PCR efficiency in amplifying both RSV and hMPV over the original primer, A1 (Figures S2 and S3 in Additional data file 1). The increased PCR efficiency contributed to increased hybridization of DNA to the probes, and is reflected in the uniformly higher signal intensities observed using primer A2. Consequently, >70% of viral probes had signal intensities above detection threshold when using primer A2, compared to approximately 20% using primer A1 (Anova test, *P *= 0.00026; Figure S3 in Additional data file 1).

### PDA: an algorithm for detecting pathogens

**Figure 5:**
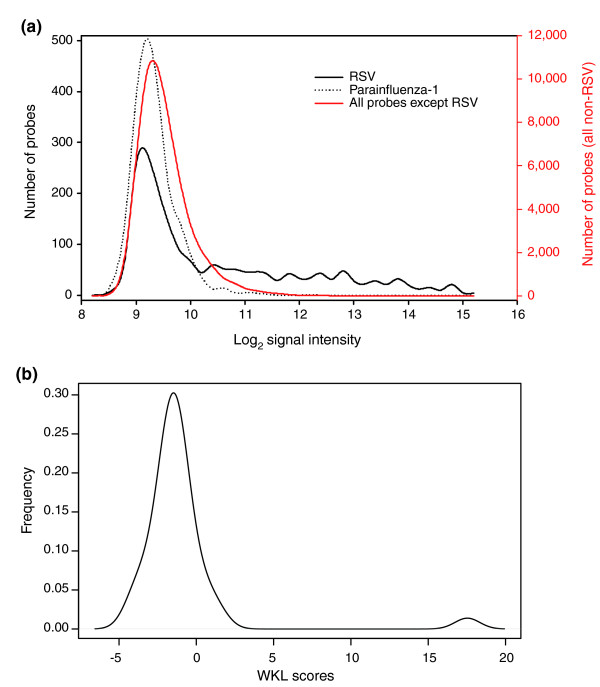
Distribution of probe signal intensities and WKL scores. RNA isolated from a RSV-infected patient was hybridized onto the array. **(a) **Distribution of probe signal intensities of all 53,555 probes (red) and r-signature probes for an absent pathogen, for example, parainfluenza-1 (dotted line), show a normal distribution. The distribution of signal intensity for RSV r-signature probes are positively skewed, with higher signal intensities in the tail of the distribution. **(b) **Distribution frequency of WKL scores for the 35 pathogen r-signatures with the majority ranging between -5 and 3. A non-normal WKL score distribution is observed (*P *< 0.05 by Anderson Darling test). The presence of a pathogen is indicated by a non-normal distribution caused by outlier WKL = 17, corresponding to RSV. Excluding the RSV r-signature WKL score results in a normal distribution. From this computation, we conclude that RSV is present in the hybridized sample.

We observed that while the signal intensities for all pathogen r-signatures approximate a normal distribution, a large proportion of probes comprising the signature of a detectable pathogen have relatively strong signal intensities resulting in a right-skewed distribution (Figure 5a). We reasoned that analysis of the tails of the signal intensity distributions for each r-signature might better enable not only the identification of an infecting pathogen, but also the presence of co-infecting pathogens in the same sample. Thus, we devised a robust statistics-based PDA that analyzes the distribution of probe signal intensities relative to the *in silico *r-signatures (see supplemental methods in Additional data file 1 and [25]). The PDA software comprises two parts: evaluation of signal intensity of probes in each pathogen r-signature using a modified Kullback-Leibler Divergence (KL); and statistical analysis of modified KL scores using the Anderson-Darling test.

Since the original KL cannot reliably determine differences in the tails of a probability distribution, and is highly dependent on the number of probes per genome and the size of each signal intensity bin, we incorporated the Anderson-Darling statistic to give more weight to the tails of each distribution. By using a cumulative distribution function instead of the original probability distribution, the *p *value generated is independent of the binning criteria, eliminating errors that occur if a particular signal intensity bin is empty [26,27]. We call our modified KL divergence the 'weighted Kullback-Leibler divergence' (WKL):

WKL(Pa|Pa¯)=∑j=0k−1Qa(j)log⁡(Qa(j)Qa¯(j))Qa¯(j)⌊1−Qa¯(j)⌋
 MathType@MTEF@5@5@+=feaafiart1ev1aaatCvAUfeBSjuyZL2yd9gzLbvyNv2Caerbhv2BYDwAHbqedmvETj2BSbqee0evGueE0jxyaibaiKI8=vI8tuQ8FMI8Gi=hEeeu0xXdbba9frFj0=OqFfea0dXdd9vqai=hGuQ8kuc9pgc9s8qqaq=dirpe0xb9q8qiLsFr0=vr0=vr0dc8meaabaqaciGacaGaaeqabaqadeqadaaakeaacaWGxbGaam4saiaadYeacaGGOaGaamiuamaaBaaaleaacaWGHbaabeaakiaacYhadaqdaaqaaiaabcfadaWgaaWcbaGaaeyyaaqabaaaaOGaaiykaiabg2da9maaqahabaWaaSaaaeaacaWGrbWaaSbaaSqaaiaadggaaeqaaOGaaiikaiaadQgacaGGPaGaciiBaiaac+gacaGGNbGaaiikamaalaaabaGaamyuamaaBaaaleaacaWGHbaabeaakiaacIcacaWGQbGaaiykaaqaaiaadgfadaWgaaWcbaWaa0aaaeaacaWGHbaaaaqabaGccaGGOaGaamOAaiaacMcaaaGaaiykaaqaamaakaaabaGaamyuamaaBaaaleaadaqdaaqaaiaadggaaaaabeaakiaacIcacaWGQbGaaiykamaagmaabaGaaGymaiabgkHiTiaadgfadaWgaaWcbaWaa0aaaeaacaWGHbaaaaqabaGccaGGOaGaamOAaiaacMcaaiaawcp+caGL7JpaaSqabaaaaaqaaiaadQgacqGH9aqpcaaIWaaabaGaam4AaiabgkHiTiaaigdaa0GaeyyeIuoaaaa@64EF@

where *Q*_*a*_(*j*) is the cumulative distribution function of the signal intensities of the probes in *P*_*a *_found in bin *b*_*j *_; Qa¯(j)
 MathType@MTEF@5@5@+=feaafiart1ev1aaatCvAUfeBSjuyZL2yd9gzLbvyNv2Caerbhv2BYDwAHbqedmvETj2BSbqee0evGueE0jxyaibaiKI8=vI8tuQ8FMI8Gi=hEeeu0xXdbba9frFj0=OqFfea0dXdd9vqai=hGuQ8kuc9pgc9s8qqaq=dirpe0xb9q8qiLsFr0=vr0=vr0dc8meaabaqaciGacaGaaeqabaqadeqadaaakeaacaWGrbWaaSbaaSqaamaanaaabaGaamyyaaaaaeqaaOGaaiikaiaadQgacaGGPaaaaa@3778@ is the cumulative distribution function of the signal intensities of the probes in Pa¯
 MathType@MTEF@5@5@+=feaafiart1ev1aaatCvAUfeBSjuyZL2yd9gzLbvyNv2Caerbhv2BYDwAHbqedmvETj2BSbqee0evGueE0jxyaibaiKI8=vI8tuQ8FMI8Gi=hEeeu0xXdbba9frFj0=OqFfea0dXdd9vqai=hGuQ8kuc9pgc9s8qqaq=dirpe0xb9q8qiLsFr0=vr0=vr0dc8meaabaqaciGacaGaaeqabaqadeqadaaakeaadaqdaaqaaiaadcfadaWgaaWcbaGaamyyaaqabaaaaaaa@3525@ found in bin *b*_*j*_. R-signatures representing absent pathogens should have normal signal intensity distributions and thus relatively low WKL scores, whereas those representing present pathogens should have high, statistically significant outlying WKL scores (Figure 5b). In the second part of PDA, the distribution of WKL scores is subjected to an Anderson-Darling test for normality. If *P *< 0.05, the WKL distribution is considered not normal, implying that the pathogen with an outlying WKL score is present. Upon identification of a pathogen, that pathogen's WKL score is left out, and a separate Anderson-Darling test is performed to test for the presence of co-infecting pathogens. In this manner, the procedure is iteratively applied until only normal distributions remain (that is, *P *> 0.05). The PDA algorithm is extremely fast, capable of making a diagnosis from a hybridized microarray in less than 10 seconds.

### Microarray performance on clinical specimens

**Figure 6:**
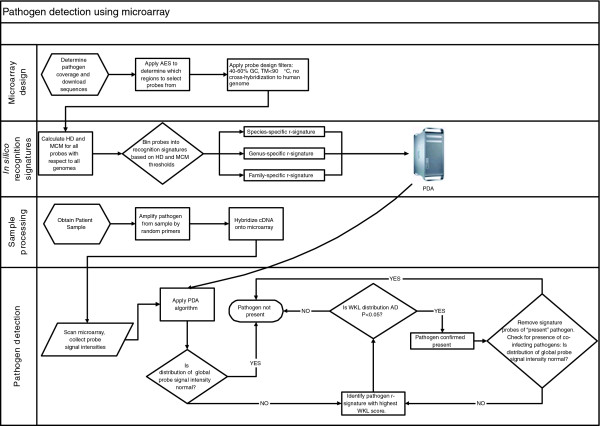
Schema of pathogen detection process. AD, Anderson-Darling.

To assess the clinical utility of the pathogen prediction platform, we analyzed 36 nasal wash specimens according to the workflow illustrated in Figure 6. These specimens were obtained from children under 4 years of age with lower respiratory tract infections (LRTI), of which 14 were hospitalized for severe disease and 22 with ambulatory LRTI. The clinical diagnosis of these patients was bronchiolitis or pneumonia. All 36 specimens had been previously analyzed for the presence of hMPV, and RSV A and B using real-time PCR. Twenty-one specimens tested positive for one or more viruses, while fifteen were PCR-negative for all three. All specimens were analyzed by microarray in a blinded fashion (Table 2).

**Table 2:** Comparison of microarray and real-time PCR performance in detection of pathogen genera (HRV, pneumovirus)

Patient ID	Array	WKL	*P *value	PDA genus diagnosis	PCR diagnosis	PCR Ct value	Virus copy no.
111	35915			ND	ND		
122	35887	20.87	2.47 × 10^-29^	Pneumovirus	Pneumovirus	24.8	5.0 × 10^4^
133	71180	22.33	6.93 × 10^-62^	Pneumovirus	Pneumovirus	25.1	4.0 × 10^4^
165	66691	16.95	3.49 × 10^-4^	Pneumovirus	Pneumovirus	27.9	3.9 × 10^3^
185*	66696			ND	ND		
254	70935	25.02	2.87 × 10^-39^	Pneumovirus	Pneumovirus	22	5.4 × 10^5^
261*	66697			ND	ND		
283*	63781	23.99	2.28 × 10^-25^	Pneumovirus	HRV	28.3	6.1 × 10^4^
		14.07	4.66 × 10^-11^	HRV			
312*	66701			ND	Pneumovirus^†^	33.7	44
321*	71006			ND	Pneumovirus^†^	31.1	340
324*	35259	20.61	3.55 × 10^-94^	Pneumovirus	Pneumovirus	21.4	3.0 × 10^6^
331*	66698			ND	HRV	31.7	3.6 × 10^3^
337	71192	21.73	3.49 × 10^-14^	Pneumovirus	Pneumovirus	26.2	1.1 × 10^5^
		8.3	1.92 × 10^-4^	HRV	HRV	29.1	3.1 × 10^4^
355	35662	18.00	2.97 × 10^-40^	Pneumovirus	Pneumovirus	20.3	6.7 × 10^6^
368*	66702			ND	ND		
374	66695			ND	Pneumovirus	34.1	500
378	70933	13.82	7.77 × 10^-17^	Pneumovirus	Pneumovirus	23.9	5.4 × 10^5^
393*	71189	25.41	1.15 × 10^-18^	HRV	HRV	30.2	2.1 × 10^5^
412	35890	19.66	2.42 × 10^-49^	Pneumovirus	Pneumovirus	23.5	6.9 × 10^5^
414	71025	49.91	1.18 × 10^-65^	Pneumovirus^†^	Pneumovirus^†^	22.3	3.9 × 10^5^
					HRV	33	2.6 × 10^3^
461	66699			ND	ND		
478	71027			ND	Pneumovirus^†^	34.8	18
483*	36053	12.17	1.47 × 10^-12^	Pneumovirus	Pneumovirus	24.8	2.9 × 10^5^
554	70997	78.55	4.59 × 10^-120^	HRV	HRV	23.5	1.5 × 10^6^
573	66700	38.09	6.26 × 10^-22^	HRV	HRV	22.2	3.6 × 10^6^
639*	71182	9.23	7.91 × 10^-6^	HRV	ND		
699	71007			ND	ND		
769	73067	24.62	3.70 × 10^-52^	Pneumovirus	Pneumovirus	25.7	2.5 × 10^4^
818	70927	10.40	1.63 × 10^-8^	HRV	HRV	34.2	1.2 × 10^3^
832	73068	13.52	4.54 × 10^-6^	Pneumovirus	Pneumovirus	28.2	3.1 × 10^3^
		40.43	1.73 × 10^-36^	Pneumovirus^†^	Pneumovirus^†^	23.8	1.2 × 10^5^
841	73070	22.11	6.80 × 10^-50^	Pneumovirus	Pneumovirus	20.9	4.5 × 10^6^
						35.4	8
					HRV	29.2	3.3 × 10^4^
853*	66690			ND	ND		
859	71188	72.17	1.42 × 10^-128^	HRV	HRV	24.5	2.8 × 10^6^
892*	68359	12.43	5.77 × 10^-5^	HRV	Pneumovirus	34	27
					HRV	32.3	4.2 × 10^3^
913	71028	40.67	1.60 × 10^-50^	Pneumovirus^†^	Pneumovirus^†^	19.1	4.7 × 10^6^
924*	66703	12.79	2.56 × 10^-6^	Pneumovirus^†^	Pneumovirus^†^	31.5	250
					Pneumovirus	33.7	630

*Hospitalized patients. ^†^RSV A patient samples. ND, none detected.

As the RSV A full-genome sequence has not been published, our array was not designed to specifically detect this virus. Thus, we first assessed array performance using only results from the 16 patients diagnosed with either hMPV or RSV B by PCR (Table 3). Of this cohort, the microarray correctly detected the presence of hMPV or RSV B in 13/16 samples. This corresponds to an assay specificity of 100%, sensitivity of 76%, and diagnostic accuracy of 94%. All 4 false negative samples (patients 374, 841, 892, and 924) had Ct values >33.5, which is near the detection limit of real-time PCR, and thus perhaps beyond the range of detection by microarray.

**Table 3:** Comparison of microarray and real-time PCR performance in detecting RSV B or hMPV

Patient ID	Array	WKL	*P *value	PDA diagnosis	PCR diagnosis	PCR Ct value	Virus copy no.
122	35887	20.87	2.47 × 10^-29^	hMPV	hMPV	24.8	5.0 × 10^4^
133	71180	22.33	6.93 × 10^-62^	hMPV	hMPV	25.1	4.0 × 10^4^
165	66691	16.95	3.49 × 10^-4^	hMPV	hMPV	27.9	3.9 × 10^3^
254	70935	25.02	2.87 × 10^-39^	hMPV	hMPV	22	5.4 × 10^5^
769	73067	24.62	3.70 × 10^-52^	hMPV	hMPV	25.7	2.5 × 10^4^
832	73068	13.52	4.54 × 10^-6^	hMPV	hMPV	28.2	3.1 × 10^3^
892*	68359			ND	hMPV	34	27
324*	35259	20.61	3.55 × 10^-94^	RSV B	RSV B	21.4	3.0 × 10^6^
355	35662	18.00	2.97 × 10^-40^	RSV B	RSV B	20.3	6.7 × 10^6^
374	66695			ND	RSV B	34.1	500
378	70933	13.82	7.77 × 10^-17^	RSV B	RSV B	23.9	5.4 × 10^5^
412	35890	19.66	2.42 × 10^-49^	RSV B	RSV B	23.5	6.9 × 10^5^
483*	36053	12.17	1.47 × 10^-12^	RSV B	RSV B	24.8	2.9 × 10^5^
924*	66703			ND	RSV B	33.7	630
337	71192	21.73	3.49 × 10^-14^	RSV B	RSV B		1.1 × 10^5^
841	73070	22.66	4.21 × 10^-50^	RSV B	RSV B	20.9	4.4 × 10^6^
					hMPV	35.4	8

*Hospitalized patients. ND, none detected.

We next assessed array performance in the group of patients PCR-positive for RSV A (*n *= 7) and PCR-negative for all tested viruses (*n *= 15). The microarray made only two positive calls in this group, both for RSV B. Interestingly, both RSV B calls corresponded to high-titre RSV A specimens by PCR (patients 414 and 913), suggesting that certain probe sets can detect the presence of related, but unspecified, viruses. Analysis of the published RSV A partial genome sequence (923 bp, Genbank ID: AF516119) revealed that 7 probes on our microarray had 100% identity to RSV A. We created an 'RSV A r-signature' comprising these 7 probes, enabling the specific detection of RSV A by microarray in 4/7 patient samples PCR-positive for RSV A (patients 414, 832, 913, and 924). Although the performance of this small r-signature was not as robust as the other virus r-signatures (median size: 249 probes), it suggested that it was feasible to pursue a 'viral discovery' approach using r-signatures created to detect viruses at the family or genus level that were related to those species already represented on the microarray. Specifically, we binned probes into family- or genus-level r-signatures by relaxing our similarity criteria (to HD ≤ 5 or MCM ≥ 25) and selecting probes common to genome sequences within families and genera for the picornaviridae family, paramyxoviridae family, rhinovirus genus (HRV) and pneumovirus genus (inclusive of RSV and hMPV).

Upon re-analysis of all 36 samples, we identified the presence of pneumovirus in 17 specimens as expected (1 false positive, patient 283), and additionally detected the presence of HRV in 9 specimens (Table 2). As HRV was a novel discovery, we re-screened all 36 samples by PCR and found HRV in 11 specimens. All nine HRV calls by microarray were confirmed by PCR except for one. This finding was intriguing given that the genomic diversity of the over 100 known rhinovirus serotypes makes detection by PCR notoriously difficult [28]. As the real-time PCR primers were capable of identifying only approximately 70% of rhinovirus strains, it is possible that the microarray correctly detected a rhinovirus strain that PCR failed to detect. Similarly, the pneumovirus genus detected in patient 283 could not be verified by RT-PCR, possibly owing to subtle genetic variations that prevented primer annealing. Thus, the greater genomic coverage afforded by the microarray might, in some cases, provide a more sensitive and accurate detection capability than pathogen-specific PCR.

Though the microarray identified the majority of HRV and RSV A samples using the genus-level r-signatures, it failed to detect three samples positive for HRV and three positive for RSV A by real-time PCR. These false negatives had an average Ct value >32, again suggesting a detection threshold close to that of real-time PCR. However, that the microarray also made a number of accurate discoveries in the 30-35 Ct range suggests a considerable degree of detection variability in the titre range above an approximately 30 Ct equivalency. Notably, the microarray correctly detected the presence of co-infecting pathogens in two samples (337 and 832), demonstrating the unique potential of this microarray platform to reveal complex disease etiologies.

### Alternative methods of array design and pathogen detection

Though pathogen detection by microarray is a young field, a number of different platforms and approaches have been described, each with important attributes. For example, the array described by Wang *et al*. [9] is based on probes designed to recognize the most conserved viral domains, facilitating the detection of a taxonomic fingerprint that provides powerful clues to viral identity with minimal probe usage. Lin *et al*. [8], on the other hand, described a probe-dense resequencing array capable of detecting a smaller set of predefined pathogens, but with higher detection specificity, including the ability to discern highly related subtypes. The microarray described herein represents a blend of these two concepts, integrating a probe tiling approach for substantial genomic coverage (though with lower probe density than a resequencing array), with a taxonomy-based strategy for binning probes into pathogen recognition signatures. Thus, our analytical output includes both family- and genus-level predictions (for r-signatures restricted to conserved probes) as well as species-specific predictions (for r-signatures composed of conserved and unique probes). Indeed, this capability allowed us to detect and accurately identify viruses in clinical samples (Table 2).

Central to pathogen prediction are the algorithms that weigh the microarray data against pre-defined recognition signatures. Unfortunately, few such algorithms exist, and only one algorithm, E-Predict, has been reported and validated [5,29,30]. E-Predict matches hybridization signatures with predicted pathogen signatures derived from the theoretical free energy of hybridization for each microarray probe. To examine the performance of E-predict on our microarray platform, we analyzed a number of samples with both E-predict and our PDA algorithm. When applied to our microarray data, E-Predict performed well, with its first prediction tending to be the correct one (Table S2 in Additional data file 1). However, for each specimen, a number of false positive calls were also made, which seemed to reflect species with considerable sequence similarity to the true infecting pathogen (Table S2 in Additional data file 1). For example, in patient sample 412, E-Predict detected RSV (the correct pathogen), but also multiple species of coronavirus (which share some sequence similarity with RSV), yet real-time PCR using pancoronavirus primers as well as primers specific for strains OC43 and 229E indicated the absence of coronavirus from this sample (Figure S4 in Additional data file 1). These false positive calls can be explained by the fact that the function of E-Predict is less geared towards identifying and distinguishing specific pathogen strains, and aimed more at elucidating the best possible candidates as supported by the available probes. Thus, E-Predict is particularly advantageous in situations where a pathogen's sequence is not fully known [5]. In contrast, our PDA algorithm is designed to make calls with greater species-level resolution. A major strength of PDA is its ability to specifically identify sequence-characterized and co-infecting pathogens with low false positivity. This is aptly demonstrated by the ability of PDA to detect specifically the presence of Dengue 1 in the clinical sample, where 7/35 viruses on the array are from the Flaviviridae family, including 4 dengue serotypes that share 70% sequence homology. The benefits of using both algorithms simultaneously for detecting both known and novel pathogens should be further evaluated.

An important discovery in this study was that the composition of the random primer tag has a significant impact on the efficiency of viral genome amplification, as assessed by an amplification efficiency score. The measurement of amplification efficiency allowed us to predict which probes would provide the most informative recognition signatures, markedly improving our pathogen prediction capability. Moreover, this finding allowed us to design AES-optimized primers that increased the amplification efficiency of our samples, resulting in greater sensitivity of pathogen detection. Whether multiplex RT-PCR using a variety of AES-designed primer tags can further increase amplification efficiency warrants further investigation. Additionally, it is feasible that other tag-based PCR applications, such as the generation of DNA libraries and enrichment of RNA for resequencing, may benefit from primer optimization using the AES algorithm.

DNA microarrays have the potential to revolutionize clinical diagnostics through their ability to simultaneously investigate thousands of potential pathogens in order to make a diagnosis. However, questions remain regarding their sensitivity and reliability. In this work, we investigated the myriad factors that influence microarray performance in the context of virus detection in clinical specimens, and describe an optimized platform capable of identifying individual and co-infecting viruses with high accuracy and sensitivity that brings microarray technology closer to the clinic. Future improvements will include significant reductions in microarray manufacturing and usage costs. Multiplex microarray formats and 're-usable' arrays are developing technologies that promise to drive down these costs. Furthermore, alternative technologies, such as beads [31], microfluidics [32,33] and nanotube microarrays [34], might provide advantages in both assay cost and speed relative to traditional microarray platforms. Technology considerations aside, the advantages of a highly parallel, nucleic acid-based screening approach for detecting disease pathogens are clear. Validations in larger patient cohorts and in diverse clinical settings will be an important next step towards establishing the clinical role of pathogen detection microarrays.

## Materials and methods

### Microarray synthesis

Complete genome sequences of 35 clinically relevant human viruses (Table S1 in Additional data file 1) were downloaded from the NCBI Taxonomy Database [35] and used to generate 40-mer probe sequences tiled across each genome and overlapping at an average 8-base resolution. Seven replicates of each probe were synthesized at random positions on the microarray using Nimblegen proprietary technology [13]. For quality control purposes, 10,000 random sequence probes with 40-60% GC content were included to assess background signal levels. Additional controls included 400 probes to human immune genes (positive controls) and 162 probes to a plant virus, PMMV (negative control). In total, 390,482 probes were synthesized on the array.

### Sample preparation, microarray hybridization and staining

Dengue (ATCC #VR-1254) was cultured as per ATCC recommendations and Sin850 SARS was cultured as described [36]. Clinical specimens (nasopharyngeal washes) were obtained from an Indonesian pediatric population using a standardized WHO protocol as described [37]. The patients were all aged between 0 and 48 months, showed symptoms of LRTI, and were diagnosed with bronchiolitis or pneumonia when they visited the clinic between February 1999 and February 2001. Of these patients, 14 were subsequently hospitalized. The samples were stored at -80°C in RNAzol (Leedo Medical Laboratories, Inc., Friendswood, TX, USA). RNA was later extracted from samples with RNAzol according to the manufacturer's instructions [38,39], resuspended in RNA storage solution (Ambion, Inc., Austin, TX, USA) and frozen at -80°C until further use. A detailed protocol is provided in the supplemental methods in Additional data file 1. Briefly, RNA was reverse transcribed to cDNA using tagged random primers as described [9,40]. The original primer A1 was 5' GTTTCCCAGTCACGATANNNNNNNNN; and the AES-optimized primer A2 was 5' GATGAGGGAAGATGGGGNNNNNNNNN. The cDNA was then amplified by random PCR, fragmented, end-labeled with biotin, hybridized onto the microarray and stained as previously described [19] with 1 exception: the addition of 0.82 M tetramethylammonium chloride (TMAC) to Nimblegen's hybridization buffer to minimize nonspecific hybridization.

### Real-time PCR for clinical samples

A 20 μl reaction mixture containing 2 μl of the purified patient RNA, 5 U of MuLV reverse transcriptase, 8 U of recombinant RNase inhibitor, 10 μl of 2X universal PCR Master Mix with no UNG (all from Applied Biosystems, Foster City, CA, USA) was combined with 0.9 μM primer and 0.2 μM (RSV B and hMPV), 0.3 μM (HRV) or 0.5 μM (RSV A) probe. The primers and probe sequences for hMPV were: 5'-AGCAAAGCAGAAAGTTTA TTCGTTAA-3'; 5'-ACCCCCCACCTCAGCATT-3'; and 5'-FAM-ATTCATGCAA GCTTATGGTGCTGGTCAAA-TAMRA-3'. Primers and probes for RSV [41] and HRV [42] have been described. Samples underwent reverse transcription at 48°C for 30 minutes, then were heated at 95°C for 10 minutes and amplified by 40 cycles of 15 s at 95°C and 1 minute at 60°C on an ABI Prism 7900HT Sequence Detection System (Applied Biosystems). During amplification, fluorescence emissions were monitored at every thermal cycle. The threshold (Ct) represents the cycle at which significant fluorescence is first detected. Ct value was converted to copy number using a control plasmid of known concentration: RSV A, 5.06 × 10^9 ^copies had a Ct value of 10.469; RSV B, 2.61 × 10^9 ^copies had a Ct value of 11.897; hMPV, 7.51 × 10^9 ^copies had a Ct value of 10.51; HRV, 1.73 × 10^7 ^copies had a Ct value of 20.20.

### One-step real-time PCR for coronavirus

Frozen live cultures of human coronavirus OC43 and 229E were purchased from ATCC (Cat #VR-1558, VR-740) for use as positive controls. RNA was extracted from these cultures using RNA Mini Kit (Qiagen, Hilden, Germany) in accordance with the manufacturer's instructions. The samples were amplified using diagnostic primer pairs for pancoronavirus, OC43 and 229E as previously described [43].

### Data analysis

Microarrays were scanned at 5 μm resolution using an Axon 4000b scanner and Genepix 4 software (Molecular Devices, Sunnyvale, CA, USA). Signal intensities were extracted using Nimblescan 2.1 software (NimbleGen Systems, Madison, WI, USA). Using an automated script (J George and V Vega), we calculated the median signal intensity and standard deviation from the seven replicates of each probe. The probe signal intensities were sorted by genome and arranged in sequence order, then reformatted into CDT format for graphical viewing of signal intensities in Java Treeview [44]. In parallel, the probe median signal intensities were analyzed using PDA to determine which pathogen was present, and the associated confidence level of prediction. The AES and PDA algorithms are described in detail in the Results section and all algorithms, formulae, software and microarray data are available on the supplemental website [25] and in Additional data file 1.

## Additional data files

The following additional data are available with the online version of this paper. Additional data file 1 includes supplementary materials and methods, figures, tables, pathogen microarray data and software.

## Supplementary Material

Additional data file 1 All files are available for download in PDF, JPG, GIF, TIFF, HTML or ZIP formats as indicated on the webpage [25]. Supplementary methods: sample amplification and microarray protocols (PDF); RT-PCR modeling and amplification efficiency score (AES); pathogen detection algorithm (PDA). Supplementary figures. Figure S1: Probe design schema. Probes (40-mers) were tiled at an average 8-base resolution across each of the 35 viral genomes in the manner depicted above. Numbers represent the start and end positions of each probe. Figure S2: Choice of primer tag in random RT-PCR has significant effect on PCR efficiency. Heatmap of probe signal intensities for a clinical hMPV sample following random RT-PCR using original primer **(a) **A1 or **(b) **AES-optimized primer A2. Figure S3: Comparison of amplification efficiency of original primer A1 and AES-optimized primer A2. RNA from patients infected with RSV B (*n *= 5) or hMPV (*n *= 3) were reverse-transcribed and amplified using primer A1 or A2 and the percentage of r-signature probes with signal above detection threshold was determined. Figure S4: Diagnostic PCR results for RSV patient 412 show that the patient does not have a coronavirus infection. **(a) **PCR using pancoronavirus primers. Lane 1, 1 kb ladder; lane 2, blank; lane 3, OC43 coronavirus positive control; lane 4, 229E coronavirus positive control; lane 5, RSV patient 412; lane 6, PCR primers and reagents only, as a negative control. **(b) **PCR using OC43 specific primers. Lane 1, 50 bp ladder; lane 2, blank; lane 3, OC43 coronavirus positive control; lane 4, RSV patient 412; lane 5, purified RSV from ATCC; lane 6, PCR negative control. **(c) **PCR using 229E specific primers. Lane 1, 229E coronavirus positive control; lane 2, RSV patient 412; lane 3, PCR negative control; lane 4, 1 kb ladder. Supplementary tables. Table S1: List of genomes represented on the pathogen detection microarray. Table S2: Comparison of E-Predict and PDA algorithms. Pathogen microarray data: data have been deposited in NCBI's Gene Expression Omnibus and are accessible through GEO accession number GSE3779 [45]. Software downloads. Amplification efficiency score software: Primerselect Readme.txt; Primerselect.java. Pathogen detection algorithm (PDA): WKL Readme.txt; WKL.cpp. Click here for file
