# Supplementary material for: Optimization and clinical validation of a pathogen detection microarray
Source: Genome Biol. 2007 May 28;8(5):R93. doi: 10.1186/gb-2007-8-5-r93 (PMC1929155; doi:10.1186/gb-2007-8-5-r93)
Supplement: Additional data file 1 — All files are available for download in PDF, JPG, GIF, TIFF, HTML or ZIP formats as indicated on the webpage [25]. Supplementary methods: sample amplification and microarray protocols (PDF); RT-PCR modeling and amplification efficiency score (AES); pathogen detection algorithm (PDA). Supplementary figures. Figure S1: Probe design schema. Probes (40-mers) were tiled at an average 8-base resolution across each of the 35 viral genomes in the manner depicted above. Numbers represent the start and end positions of each probe. Figure S2: Choice of primer tag in random RT-PCR has significant effect on PCR efficiency. Heatmap of probe signal intensities for a clinical hMPV sample following random RT-PCR using original primer (a) A1 or (b) AES-optimized primer A2. Figure S3: Comparison of amplification efficiency of original primer A1 and AES-optimized primer A2. RNA from patients infected with RSV B (n = 5) or hMPV (n = 3) were reverse-transcribed and amplified using primer A1 or A2 and the percentage of r-signature probes with signal above detection threshold was determined. Figure S4: Diagnostic PCR results for RSV patient 412 show that the patient does not have a coronavirus infection. (a) PCR using pancoronavirus primers. Lane 1, 1 kb ladder; lane 2, blank; lane 3, OC43 coronavirus positive control; lane 4, 229E coronavirus positive control; lane 5, RSV patient 412; lane 6, PCR primers and reagents only, as a negative control. (b) PCR using OC43 specific primers. Lane 1, 50 bp ladder; lane 2, blank; lane 3, OC43 coronavirus positive control; lane 4, RSV patient 412; lane 5, purified RSV from ATCC; lane 6, PCR negative control. (c) PCR using 229E specific primers. Lane 1, 229E coronavirus positive control; lane 2, RSV patient 412; lane 3, PCR negative control; lane 4, 1 kb ladder. Supplementary tables. Table S1: List of genomes represented on the pathogen detection microarray. Table S2: Comparison of E-Predict and PDA algorithms. Pathogen microarray data: data have been [file gb-2007-8-5-r93-S1.zip › Documents and Settings/wongc/My Documents/Presentations/My publications/Current paper/Genome Biology/Genome Biology website/index.htm]

Optimizing Pathogen Chip


# Optimizing Microarray Platform for the Detection of Unknown Pathogens Directly from Human Tissue Samples

*Christopher W. Wong1,
Charlie Lee Wah Heng2, Leong Wan Yee1, Shirlena Soh3,* *Cissy B.
Kartasasmita4, Eric A. F. Simoes5, Martin L. Hibberd3, Ken W-K Sung2 and Lance D. Miller1*

*1*Microarray & Expression Genomics, *2*Information
& Mathematical Sciences, *3*Infectious Disease Laboratories, Genome Institute of
Singapore, SINGAPORE; *4*Department of Pediatrics, Faculty of Medicine,
Universitas Padjadjaran, INDONESIA; *5*Section of Infectious
Diseases, The University of Colorado School of Medicine and The Children’s
Hospital, Denver, CO, USA.

---

This
page contains supplementary information for the paper of the same name
published in xxxxx.

[pdf]

- Supplementary
  Methods
- Supplementary
  Figures
- Supplementary
  Tables
- Pathogen Microarray
  Data
- Software downloads

## 

## Supplementary Methods

1.      Sample
Amplification and Microarray Protocols [PDF]

2.      RT-PCR
Modeling and Amplification Efficiency Score (AES) [PDF]

3.      Pathogen
Detection Algorithm (PDA) [PDF]

## 

## Supplementary Figures

**Figure S1.**  Probe design schema.  Probes (40-mers) were tiled at an
average 8-base resolution across each of the 35 viral genomes in the manner
depicted above. Numbers represent the start and end positions of each
probe.  [JPG]

**Figure S2.**  Choice
of primer tag in random RT-PCR has significant effect on PCR efficiency.  (A) Heatmap of probe signal intensities
for a clinical hMPV sample following random RT-PCR using original primer A1 or
(B) AES-optimized primer A2.  [GIF]  [TIFF]

**Figure S3.**  Comparison of amplification efficiency
of original primer A1 and AES-optimized primer A2.  RNA from patients infected with RSV B
(n=5) or hMPV (n=3) were reverse-transcribed and amplified using primer A1 or
A2 and the percentage of r-signature probes with signal above detection
threshold was determined.  [JPG]

**Figure S4.**  Diagnostic PCR results for RSV Patient
#412 show that patient does not have a coronavirus infection.  (A) PCR using Pancoronavirus
primers.  Lane 1: 1 kb ladder, Lane
2: blank, Lane 3: OC43 coronavirus positive control, Lane 4: 229E coronavirus
positive control, Lane 5: RSV patient #412, Lane 6: PCR primers and reagents
only, as a negative control.  (B)
PCR using OC43 specific primers. 
Lane 1: 50 bp ladder, Lane 2:
blank, Lane 3: OC43 coronavirus positive control, Lane 4: RSV patient #412,
Lane 5: purified RSV from ATCC, Lane 6: PCR negative control.  (C) PCR using 229E specific
primers.  Lane 1: 229E coronavirus
positive control, Lane 2: RSV patient #412, Lane 3: PCR negative control, Lane
4: 1 kb ladder. [JPG]

## 

## Supplementary Tables

**Table S1.**  List of genomes represented on the
pathogen detection microarray.  [HTML]

**Table S2.**  Comparison of E-Predict and PDA
algorithms. [HTML]

## 

## Pathogen Array Data

Files may be opened using Microsoft
Excel.  In each data file, the 1st
column represents probe ID, signal intensities for each replicate in columns
v2-v8, followed by median signal intensity and log2-transformed
median signal intensity.

·       
Download
zip data file of all arrays described in the paper (27 MB) [ZIP]

## 

## Software Downloads

Amplification
Efficiency Score software:

·       
Primerselect Readme.txt           [download]

·       
Primerselect.java                      [download]

 

Pathogen
Detection Algorithm (PDA):

·       
WKL Readme.txt                     [download]

·       
WKL.cpp                                [download]

 

**Contact authors at
wongc@gis.a-star.edu.sg** 

Last updated 2.22.2007
