# Supplementary material for: Optimization and clinical validation of a pathogen detection microarray
Source: Genome Biol. 2007 May 28;8(5):R93. doi: 10.1186/gb-2007-8-5-r93 (PMC1929155; doi:10.1186/gb-2007-8-5-r93)
Supplement: Additional data file 1 — All files are available for download in PDF, JPG, GIF, TIFF, HTML or ZIP formats as indicated on the webpage [25]. Supplementary methods: sample amplification and microarray protocols (PDF); RT-PCR modeling and amplification efficiency score (AES); pathogen detection algorithm (PDA). Supplementary figures. Figure S1: Probe design schema. Probes (40-mers) were tiled at an average 8-base resolution across each of the 35 viral genomes in the manner depicted above. Numbers represent the start and end positions of each probe. Figure S2: Choice of primer tag in random RT-PCR has significant effect on PCR efficiency. Heatmap of probe signal intensities for a clinical hMPV sample following random RT-PCR using original primer (a) A1 or (b) AES-optimized primer A2. Figure S3: Comparison of amplification efficiency of original primer A1 and AES-optimized primer A2. RNA from patients infected with RSV B (n = 5) or hMPV (n = 3) were reverse-transcribed and amplified using primer A1 or A2 and the percentage of r-signature probes with signal above detection threshold was determined. Figure S4: Diagnostic PCR results for RSV patient 412 show that the patient does not have a coronavirus infection. (a) PCR using pancoronavirus primers. Lane 1, 1 kb ladder; lane 2, blank; lane 3, OC43 coronavirus positive control; lane 4, 229E coronavirus positive control; lane 5, RSV patient 412; lane 6, PCR primers and reagents only, as a negative control. (b) PCR using OC43 specific primers. Lane 1, 50 bp ladder; lane 2, blank; lane 3, OC43 coronavirus positive control; lane 4, RSV patient 412; lane 5, purified RSV from ATCC; lane 6, PCR negative control. (c) PCR using 229E specific primers. Lane 1, 229E coronavirus positive control; lane 2, RSV patient 412; lane 3, PCR negative control; lane 4, 1 kb ladder. Supplementary tables. Table S1: List of genomes represented on the pathogen detection microarray. Table S2: Comparison of E-Predict and PDA algorithms. Pathogen microarray data: data have been [file gb-2007-8-5-r93-S1.zip › Documents and Settings/wongc/My Documents/Presentations/My publications/Current paper/Genome Biology/Genome Biology website/protocol.pdf]

# Pathogen Chip Sample Amplification and Array Hybridization Protocol

Primer A2: 5' GATGAGGGAAGATGGGGNNNNNNNNN
Primer B2: 5' GATGAGGGAAGATGGGG

## Round A: 1st strand synthesis with RT

Mix 2 µl RNA (1:10) with 2 µl primer "A2" (20 µM stock) to a final volume of 10 µl
Heat to 65 °C /5 mins
Cool at room temperature (25 °C)/5 mins
Add 10 µl of 2× enzyme mix

2× enzyme mix:

- 2.0 µl 10× RT Buffer (Stratagene)
- 1.0 µl 10 mM dNTP mix (final concentration 500 µM each nucleotide)
- 3.0 µl $H_2O$
- 2.0 µl 0.1 mM DTT
- 2.0 µl Stratascript Reverse Transcriptase (Stratagene)

Incubate at 42 °C/30 mins
Heat to 65 °C /5 mins
Cool at room temperature (25 °C)/5 mins
Add 1 µl RT
Incubate additional 42 °C /30 mins
Hold at 4 °C

## 2nd strand synthesis with Sequenase

Heat sample to 94 °C /2 mins
Rapidly cool to 10 °C, hold at 10 °C /5 mins
Add 10 µl Sequenase mix for a total RXN volume of 30 µl

Sequenase Mix:

- 2.0 µl 5× Sequenase Buffer
- 7.7 µl $H_2O$
- 0.3 µl Sequenase (USB)

Ramp from 10 °C to 37°C over 8 mins, hold at 37 °C /8 mins
Rapid ramp to 94 °C, hold at 94 °C /2 mins
Rapid ramp to 10 °C , hold at 10 °C /5 mins while adding 1.2 µl of diluted Sequenase (1:4 dilution)
Ramp from 10 °C to 37 °C over 8 mins, hold at 37 °C/8mins
Hold at room temperature
Sample total volume = 30 µl

## Round B: PCR amplification

| | |
|---|---|
| Round A Template | 30 µl |
| 10× PCR Buffer | 10 |
| 50 mM $MgCl_2$ | 4 |
| 10 mM dNTP | 2.5 |
| Primer B2 (100 µM stock) | 1 |
| Platinum *Taq* (Invitrogen) | 1 |
| $ddH_2O$ | 51.5 |

Round B cycles:
Ramp to 94 °C C, hold at 94 °C C/8 mins
94 °C C/30 secs
40 °C C/30 secs } 40×
50 °C C/30 secs
72 °C C/1 min
4 °C C/∞
Each PCR product total volume = 100 µl
Loaded 5 µl into 1% agarose gel before proceed on to concentration (to 6 µl).

## Digestion of Sample:

Make up 0.1U/ µl DNase I:

| | |
|---|---|
| Stock 5U/ µl DNase I | 2 µl |
| $ddH_2O$ | 98 µl |

Make up Digestion mix:

| | | |
|---|---|---|
| 10× One-Phor-All Buffer | 1 µl | good for 1 array |
| 0.1 U/µl DNase I | 1 µl | |
| $ddH_2O$ | 2 µl | |
| TOTAL | 4 µl | |

For each sample:

| | |
|---|---|
| Round B DNA | 6 µl |
| Digestion mix | 4 µl |

Incubate at 37 °C /3 mins
Incubate at 97 °C /15 mins; spin down each 5 mins or so
Place on ice for at least 3 mins

## Biotin labeling:

| | |
|---|---|
| Digested product | 10 µl |
| Biotin-N6-ddATP | 1 µl |
| Terminal transferase | 2 µl |

Incubate at 37 °C /90 mins (can start prehyb after 1 hr)
Incubate at 97 °C /15 mins; spin down each 5 mins or so

## Preparation of prehybridization and hybridization solutions:

For each microarray being hybridized, prepare the following 2 solutions in separate 1.5 ml microcentrifuge tubes.
*Note: If sample volume is less than 13 µl, top-up with ddH$_2$O.*

| Component | Prehyb solution (µl) | Hyb solution (µl) |
|---|---|---|
| Fragmented PCR products | -- | 13 |
| CPK6 Oligo (100 nM) | -- | 0.45 |
| Herring sperm DNA (10 mg/ml) | -- | 1 |
| 2× TMAC Resequencing buffer (Nimblegen proprietary) | 100 | 16 |
| 5 M TMAC | -- | 6 |
| ddH$_2$O | 100 | -- |
| TOTAL | 200 | 36.45 |

## Prehybridization:

1. Transfer prehybridization solution to 95 °C heat block/5 mins
2. Incubate at 45 °C/5 mins (at MAUI station)
3. Spin at 13000 rpm/5 mins (check for precipitation)
4. Pipet 200 µl of prehybridization solution to the array. Avoid forming bubbles. Place hybrislip cover
5. Incubate slide in MAUI chamber at 45 °C/ 20 mins
6. During this incubation, the hybridization solution can be prepared for use as described in the next section.
7. Remove hybri-strip from slide while immersed in ddH$_2$O
8. Wash in ddH$_2$O/1 min
9. Wash in 70% ethanol/1 min
10. Dry by centrifuging at 100 ×g/4 mins

## Hybridization:

1. Transfer hybridization solution to 95 °C heat block/5 mins
2. Incubate at 45 °C/5 mins (at MAUI station)
3. Spin at 13000 rpm/5 mins (check for precipitation)
4. Transfer to 45 °C heat block until prehybridization step has been completed
5. Adhere MAUI cover to slide and place inside MAUI hyb station
6. Spin briefly to collect sample and pipette 36.45 µl of sample into one of the two MAUI cover holes
7. Dry any sample leakage around the holes
8. Adhere MAUI stickers to both holes
9. Click down the fastener and start hybridization (program A)
10. Hybridize overnight (~16.5 hrs: 5.30pm- 10.00am)

## Washes and Staining:

Prepare all washes, stains and antibody amplification mixes prior to removing chip from MAUI

Wash 1 (dunk bath) – 2× SSC/0.1% SDS
Wash 2 – 0.2× SSC
Wash 3 – 0.05× SSC
Wash 4 – 70% ethanol
2× Stain buffer – put in 42 °C water bath 15 mins prior stain preparation

1× Stain solution (100 mM MES, 1M [$Na^+$], 0.05% Tween-20):

| | |
|---|---|
| 2× Stain buffer | 1500 µl |
| $ddH_2O$ (non DEPC-treated) | 1410 µl |
| Non-acetylated BSA (100 mg/ml) | 60 µl |
| Cy3-Streptavidin (1 µg/µl) | 30 µl |
| TOTAL | 3000 µl |

1× Antibody amplification solution:

| | |
|---|---|
| 2× Stain buffer | 1500 µl |
| Non-acetylated BSA (100 mg/ml) | 96 µl |
| Goat IgG (50 mg/ml) | 8.64 µl |
| Biotinylated Goat anti-SA (0.5 mg/ml) | 28.8 µl |
| $ddH_2O$ (non DEPC-treated) | 1367 µl |
| TOTAL | 3000 µl |

Dunk stain solution:

| | |
|---|---|
| 2× Stain buffer | 22.5 ml |
| $ddH_2O$ (non DEPC-treated) | 22.5 ml |
| 1× Stain solution | 3 ml |
| TOTAL | 48 ml |

Dunk antibody amplification solution:

| | |
|---|---|
| 2× Stain buffer | 22.5 ml |
| $ddH_2O$ (non DEPC-treated) | 22.5 ml |
| 1× Antibody amplification solution | 3 ml |
| TOTAL | 48 ml |

* Preparation of Goat IgG (50 mg/ml)
1 ml 150 mM NaCl + 50 mg of Goat IgG

Preparation of 150 mM NaCl
225 µl 1 M NaCl + 1275 µl $ddH_2O$

1. Stop MAUI, open fastener, remove chip with MAUI lid still attached.
2. Remove MAUI lid from array while immersed in Wash 1. Using 2 hands crack the cover off the slide using slide edge.
3. Transfer to Wash 2 for 1 min

4. Remove each slide from Wash 2. Place into stain solution (3 arrays/coplin jar of 48 ml solution). Let stand in dark for 10 mins. Invert jar to mix 3× over 10 mins.
5. Handling one slide at a time, transfer each slide back into Wash 2 for a quick wash (reuse from step 3)/1 min.
6. Place into antibody amplification solution (3 arrays/coplin jar of 48 ml solution).
7. Let stand in dark for 10 mins. Invert jar to mix 3× over 10 mins.
8. Handling one slide at a time, transfer each slide back into Wash 2 for a quick wash (reuse from step 3)/1 min.
9. Place into stain solution (3 arrays/coplin jar of 48 ml solution). Let stand in dark for 10 mins. Invert jar to mix 3× over 10 mins.
10. Transfer the slides to Wash 2/ 1 mins (new wash)
11. Transfer to Wash 3/ 30".
12. Dunk 6× in 70% ethanol.
13. Dry array by centrifuging 100 ×g/4 mins
14. Scan array on Axon scanner (within 6 hrs of wash) at 400 PMT, 5 µm resolution, no averaging (using GenePix Pro 4 software)
