# Supplementary material for: Optimization and clinical validation of a pathogen detection microarray
Source: Genome Biol. 2007 May 28;8(5):R93. doi: 10.1186/gb-2007-8-5-r93 (PMC1929155; doi:10.1186/gb-2007-8-5-r93)
Supplement: Additional data file 1 — All files are available for download in PDF, JPG, GIF, TIFF, HTML or ZIP formats as indicated on the webpage [25]. Supplementary methods: sample amplification and microarray protocols (PDF); RT-PCR modeling and amplification efficiency score (AES); pathogen detection algorithm (PDA). Supplementary figures. Figure S1: Probe design schema. Probes (40-mers) were tiled at an average 8-base resolution across each of the 35 viral genomes in the manner depicted above. Numbers represent the start and end positions of each probe. Figure S2: Choice of primer tag in random RT-PCR has significant effect on PCR efficiency. Heatmap of probe signal intensities for a clinical hMPV sample following random RT-PCR using original primer (a) A1 or (b) AES-optimized primer A2. Figure S3: Comparison of amplification efficiency of original primer A1 and AES-optimized primer A2. RNA from patients infected with RSV B (n = 5) or hMPV (n = 3) were reverse-transcribed and amplified using primer A1 or A2 and the percentage of r-signature probes with signal above detection threshold was determined. Figure S4: Diagnostic PCR results for RSV patient 412 show that the patient does not have a coronavirus infection. (a) PCR using pancoronavirus primers. Lane 1, 1 kb ladder; lane 2, blank; lane 3, OC43 coronavirus positive control; lane 4, 229E coronavirus positive control; lane 5, RSV patient 412; lane 6, PCR primers and reagents only, as a negative control. (b) PCR using OC43 specific primers. Lane 1, 50 bp ladder; lane 2, blank; lane 3, OC43 coronavirus positive control; lane 4, RSV patient 412; lane 5, purified RSV from ATCC; lane 6, PCR negative control. (c) PCR using 229E specific primers. Lane 1, 229E coronavirus positive control; lane 2, RSV patient 412; lane 3, PCR negative control; lane 4, 1 kb ladder. Supplementary tables. Table S1: List of genomes represented on the pathogen detection microarray. Table S2: Comparison of E-Predict and PDA algorithms. Pathogen microarray data: data have been [file gb-2007-8-5-r93-S1.zip › Documents and Settings/wongc/My Documents/Presentations/My publications/Current paper/Genome Biology/Genome Biology website/tableS1.htm]

| Genome | NCBI no. | Ref type | Accession no. | Description |
| 1 | 9629198 | RefSeq | NC\_001781.1 | Human respiratory syncytial virus, complete genome |
| 2 | 19718363 | RefSeq | NC\_003461.1 | Human parainfluenza virus 1 strain Washington/1964, complete genome |
| 3 | 19525721 | RefSeq | NC\_003443.1 | Human parainfluenza virus 2, complete genome |
| 4 | 10937870 | RefSeq | NC\_001796.2 | Human parainfluenza virus 3, complete genome |
| 5 | 30468042 | Genbank | AY283794.1 | SARS coronavirus Sin2500, complete genome |
| 6 | 38018022 | RefSeq | NC\_005147.1 | Human coronavirus OC43, complete genome |
| 7 | 12175745 | RefSeq | NC\_002645.1 | Human coronavirus 229E, complete genome |
| 8 | 46852132 | RefSeq | NC\_004148.2 | Human metapneumovirus, complete genome |
| 9 | 8486138 | RefSeq | NC\_002023.1 | Influenza A virus RNA segment 1, complete sequence |
|  | 8486136 | RefSeq | NC\_002022.1 | Influenza A virus RNA segment 3, complete sequence |
|  | 8486134 | RefSeq | NC\_002021.1 | Influenza A virus RNA segment 2, complete sequence |
|  | 8486131 | RefSeq | NC\_002020.1 | Influenza A virus RNA segment 8, complete sequence |
|  | 8486129 | RefSeq | NC\_002019.1 | Influenza A virus RNA segment 5, complete sequence |
|  | 8486127 | RefSeq | NC\_002018.1 | Influenza A virus RNA segment 6, complete sequence |
|  | 8486125 | RefSeq | NC\_002017.1 | Influenza A virus RNA segment 4, complete sequence |
| 10 | 8486164 | RefSeq | NC\_002204.1 | Influenza B virus RNA-1, complete sequence |
|  | 8486148 | RefSeq | NC\_002205.1 | Influenza B virus RNA-2, complete sequence |
|  | 8486150 | RefSeq | NC\_002206.1 | Influenza B virus RNA-3, complete sequence |
|  | 8486152 | RefSeq | NC\_002207.1 | Influenza B virus RNA-4, complete sequence |
|  | 8486154 | RefSeq | NC\_002208.1 | Influenza B virus RNA-5, complete sequence |
|  | 8486156 | RefSeq | NC\_002209.1 | Influenza B virus RNA-6, complete sequence |
|  | 8486159 | RefSeq | NC\_002210.1 | Influenza B virus RNA-7, complete sequence |
|  | 8486161 | RefSeq | NC\_002211.1 | Influenza B virus RNA-8, complete sequence |
| 11 | 11528013 | RefSeq | NC\_001563.2 | West Nile virus, complete genome |
| 12 | 9627244 | RefSeq | NC\_002031.1 | Yellow fever virus, complete genome |
| 13 | 13559808 | RefSeq | NC\_002728.1 | Nipah virus, complete genome |
| 14 | 11545722 | RefSeq | NC\_002617.1 | Newcastle disease virus, complete genome |
| 15 | 9629357 | RefSeq | NC\_001802.1 | Human immunodeficiency virus 1, complete genome |
| 16 | 21326584 | RefSeq | NC\_003977.1 | Hepatitis B virus, complete genome |
| 17 | 9627257 | RefSeq | NC\_001576.1 | Human papillomavirus type 10, complete genome |
| 18 | 10445391 | RefSeq | NC\_002554.1 | Foot-and-mouth disease virus C, complete genome |
| 19 | 9790308 | RefSeq | NC\_001545.1 | Rubella virus, complete genome |
| 20 | 9626732 | RefSeq | NC\_001489.1 | Hepatitis A virus, complete genome |
| 21 | 38371716 | RefSeq | NC\_005222.1 | Hantaan virus, complete genome |
| 22 | 38371727 | RefSeq | NC\_005217.1 | Sin Nombre virus, complete genome |
| 23 | 23334588 | RefSeq | NC\_004294.1 | Lymphocytic choriomeningitis virus segment S, complete sequence |
|  | 23334585 | RefSeq | NC\_004291.1 | Lymphocytic choriomeningitis virus segment L, complete sequence |
| 24 | 9626460 | RefSeq | NC\_001437.1 | Japanese encephalitis virus, genome |
| 25 | 51850386 | DNA Database of Japan | AB189128.1 | Dengue virus type 3 genomic RNA, complete genome, strain: 98902890 DF DV-3 |
| 26 | 12659201 | Genbank | AF326573.1 | Dengue virus type 4 strain 814669, complete genome |
| 27 | 19744844 | Genbank | AF489932.1 | Dengue Virus Type 2 strain BR64022, complete genome |
| 28 | 323660 | Genbank | M87512.1 | DENT1SEQ Dengue virus type 1 complete genome |
| 29 | 9626436 | RefSeq | NC\_001430.1 | Human enterovirus D, complete genome |
| 30 | 9626433 | RefSeq | NC\_001428.1 | Human enterovirus C, complete genome |
| 31 | 9627719 | RefSeq | NC\_001612.1 | Human enterovirus A, complete genome |
| 32 | 21363125 | RefSeq | NC\_003986.1 | Human echovirus 1, complete genome |
| 33 | 9626677 | RefSeq | NC\_001472.1 | Human enterovirus B, complete genome |
| 34 | 9627730 | RefSeq | NC\_001617.1 | Human rhinovirus 89, complete genome |
| 35 | 9626735 | RefSeq | NC\_001490.1 | Human rhinovirus B, complete genome |
|  |  |  |  |  |
