# Supplementary material for: Optimization and clinical validation of a pathogen detection microarray
Source: Genome Biol. 2007 May 28;8(5):R93. doi: 10.1186/gb-2007-8-5-r93 (PMC1929155; doi:10.1186/gb-2007-8-5-r93)
Supplement: Additional data file 1 — All files are available for download in PDF, JPG, GIF, TIFF, HTML or ZIP formats as indicated on the webpage [25]. Supplementary methods: sample amplification and microarray protocols (PDF); RT-PCR modeling and amplification efficiency score (AES); pathogen detection algorithm (PDA). Supplementary figures. Figure S1: Probe design schema. Probes (40-mers) were tiled at an average 8-base resolution across each of the 35 viral genomes in the manner depicted above. Numbers represent the start and end positions of each probe. Figure S2: Choice of primer tag in random RT-PCR has significant effect on PCR efficiency. Heatmap of probe signal intensities for a clinical hMPV sample following random RT-PCR using original primer (a) A1 or (b) AES-optimized primer A2. Figure S3: Comparison of amplification efficiency of original primer A1 and AES-optimized primer A2. RNA from patients infected with RSV B (n = 5) or hMPV (n = 3) were reverse-transcribed and amplified using primer A1 or A2 and the percentage of r-signature probes with signal above detection threshold was determined. Figure S4: Diagnostic PCR results for RSV patient 412 show that the patient does not have a coronavirus infection. (a) PCR using pancoronavirus primers. Lane 1, 1 kb ladder; lane 2, blank; lane 3, OC43 coronavirus positive control; lane 4, 229E coronavirus positive control; lane 5, RSV patient 412; lane 6, PCR primers and reagents only, as a negative control. (b) PCR using OC43 specific primers. Lane 1, 50 bp ladder; lane 2, blank; lane 3, OC43 coronavirus positive control; lane 4, RSV patient 412; lane 5, purified RSV from ATCC; lane 6, PCR negative control. (c) PCR using 229E specific primers. Lane 1, 229E coronavirus positive control; lane 2, RSV patient 412; lane 3, PCR negative control; lane 4, 1 kb ladder. Supplementary tables. Table S1: List of genomes represented on the pathogen detection microarray. Table S2: Comparison of E-Predict and PDA algorithms. Pathogen microarray data: data have been [file gb-2007-8-5-r93-S1.zip › Documents and Settings/wongc/My Documents/Presentations/My publications/Current paper/Genome Biology/Genome Biology website/tableS2.htm]

| Array | Patient | E-Predict algorithm | | | GISPathogen algorithm | |  |  |  |  |  |  |
|  |  | Genome | Similarity\_Score | P-value | Genome� | WKL |  | | | | | |
| 36042 | 412 (RSV) | RSV | 0.35128 | 0 | RSV | 17.531234 |  |  | | | | |
|  |  | OC43 coronavirus | 0.350264 | 6.84E-20 |  |  |  | | | | | |
|  |  | 229E coronavirus | 0.323503 | 1.77E-10 |  |  |  | | | | | |
|  |  | Hepatitis B | 0.134825 | 3.03E-04 |  |  |  | | | | | |
|  |  | SARS coronavirus | 0.338911 | 0.00299 |  |  |  | | | | | |
|  |  | Hepatitis A | 0.229589 | 0.00847 |  |  |  | | | | | |
| 36731 | 412 (RSV) | RSV | 0.335389 | 0 | RSV | 16.953261 |  | | | | | |
|  |  | OC43 coronavirus | 0.348043 | 2.29E-13 |  |  |  | | | | | |
|  |  | 229E coronavirus | 0.322055 | 2.00E-09 |  |  |  | | | | | |
|  |  | Hepatitis B | 0.135222 | 1.02E-06 |  |  |  | | | | | |
|  |  | Rubella | 0.164332 | 0.00919 |  |  |  | | | | | |
| 35890 | 412 (RSV) | RSV | 0.334602 | 0 | RSV | 17.214556 |  | | | | | |
|  |  | OC43 coronavirus | 0.348969 | 3.63E-23 |  |  |  | | | | | |
|  |  | 229E coronavirus | 0.322805 | 3.20E-14 |  |  |  | | | | | |
|  |  | Hepatitis B | 0.13436 | 6.74E-04 |  |  |  | | | | | |
|  |  | SARS coronavirus | 0.338609 | 0.03060 |  |  |  | | | | | |
| 35179 | 122 (hMPV) | hMPV | 0.260110695 | 5.01E-28 | hMPV | 9.763149 |  | | | | | |
|  |  | Rubella | 0.164784981 | 1.20E-17 |  |  |  | | | | | |
|  |  | Foot-and-mouth� C | 0.206747816 | 4.66E-11 |  |  |  | | | | | |
|  |  | Jap encephalitis | 0.201347222 | 1.65E-04 |  |  |  | | | | | |
|  |  | Hepatitis B | 0.133407622 | 1.98E-04 |  |  |  | | | | | |
|  |  | Yellow Fever | 0.200500564 | 0.00567 |  |  |  | | | | | |
|  |  | Echovirus 1 | 0.222002025 | 0.01740 |  |  |  | | | | | |
|  |  | Newcastle | 0.234481686 | 0.01820 |  |  |  | | | | | |
| 35887 | 122 (hMPV) | hMPV | 0.299655 | 0 | hMPV | 39.677149 |  | | | | | |
|  |  | Rubella | 0.169626 | 3.40E-19 |  |  |  | | | | | |
|  |  | Hepatitis B | 0.137703 | 5.84E-12 |  |  |  | | | | | |
|  |  | OC43 coronavirus | 0.347685 | 5.06E-10 |  |  |  | | | | | |
|  |  | 229E coronavirus | 0.321702 | 1.72E-06 |  |  |  | | | | | |
|  |  | SARS coronavirus | 0.340504 | 1.76E-06 |  |  |  | | | | | |
|  |  | Foot-and-mouth� C | 0.2075 | 1.31E-04 |  |  |  | | | | | |
|  |  | Newcastle | 0.23453 | 0.04310 |  |  |  | | | | | |
|  |  |  |  |  |  |  | | | | | | |
|  |  |  |  |  |  |  | | | | | | |
|  |  |  |  |  |  |  |  |  |  |  |  |  |
