# Supplementary material for: Optimization and clinical validation of a pathogen detection microarray
Source: Genome Biol. 2007 May 28;8(5):R93. doi: 10.1186/gb-2007-8-5-r93 (PMC1929155; doi:10.1186/gb-2007-8-5-r93)
Supplement: Additional data file 1 — All files are available for download in PDF, JPG, GIF, TIFF, HTML or ZIP formats as indicated on the webpage [25]. Supplementary methods: sample amplification and microarray protocols (PDF); RT-PCR modeling and amplification efficiency score (AES); pathogen detection algorithm (PDA). Supplementary figures. Figure S1: Probe design schema. Probes (40-mers) were tiled at an average 8-base resolution across each of the 35 viral genomes in the manner depicted above. Numbers represent the start and end positions of each probe. Figure S2: Choice of primer tag in random RT-PCR has significant effect on PCR efficiency. Heatmap of probe signal intensities for a clinical hMPV sample following random RT-PCR using original primer (a) A1 or (b) AES-optimized primer A2. Figure S3: Comparison of amplification efficiency of original primer A1 and AES-optimized primer A2. RNA from patients infected with RSV B (n = 5) or hMPV (n = 3) were reverse-transcribed and amplified using primer A1 or A2 and the percentage of r-signature probes with signal above detection threshold was determined. Figure S4: Diagnostic PCR results for RSV patient 412 show that the patient does not have a coronavirus infection. (a) PCR using pancoronavirus primers. Lane 1, 1 kb ladder; lane 2, blank; lane 3, OC43 coronavirus positive control; lane 4, 229E coronavirus positive control; lane 5, RSV patient 412; lane 6, PCR primers and reagents only, as a negative control. (b) PCR using OC43 specific primers. Lane 1, 50 bp ladder; lane 2, blank; lane 3, OC43 coronavirus positive control; lane 4, RSV patient 412; lane 5, purified RSV from ATCC; lane 6, PCR negative control. (c) PCR using 229E specific primers. Lane 1, 229E coronavirus positive control; lane 2, RSV patient 412; lane 3, PCR negative control; lane 4, 1 kb ladder. Supplementary tables. Table S1: List of genomes represented on the pathogen detection microarray. Table S2: Comparison of E-Predict and PDA algorithms. Pathogen microarray data: data have been [file gb-2007-8-5-r93-S1.zip › Documents and Settings/wongc/My Documents/Presentations/My publications/Current paper/Genome Biology/Genome Biology website/WKL.pdf]

Analysis of Pathogen Microarray Data

Our Pathogen Microarray contains a set of 40-mer probes $P = \{p_1, p_2, \ldots, p_s\}$, binned into distinct pathogen r-signatures for each of 35 viral genomes $V = \{v_1, v_2, \ldots, v_{35}\}$. Upon hybridization of pathogen nucleic acids, a set of probe signal intensity data $D = \{d_1, d_2, \ldots, d_s\}$ corresponding to probe set P is generated.

PDA

PDA comprises a series of statistical tests, beginning with a Weighted Kullback-Leibler test and Z-score transformation (WKL score) followed by Anderson-Darling test for normality.

Consider the virus v_a. Let P_a be the set of probes of a virus v_a and $\overline{P_a} = P - P_a$. Let $[r_{low}, r_{high}]$ be the signal intensity range. We partition it into c bins $[\ r_{low} + j(\frac{r_{high} - r_{low}}{c})\ ,\ r_{low} + (j+1)(\frac{r_{high} - r_{low}}{c})\ ]$ for j=0, 1, …, c-1. The unmodified Kullback-Leibler divergence can be computed by

$$KL(P_a \mid \overline{\mathrm{P_a}}) = \sum_{j=0}^{c-1} f_a(j) \log(\frac{f_a(j)}{f_{\bar{a}}(j)})$$

where n_a^j and $n_{\bar{a}}^j$ are the number of probes in P_a and probes in $\overline{P_a}$ contained in the bin b_j respectively. $f_a(j) = \frac{n_a^j}{\sum_{h=0}^{c-1} n_a^h}$ is the fraction of probes in P_a found in bin b_j; and

$f_{\bar{a}}(j) = \frac{n_{\bar{a}}^j}{\sum_{h=0}^{c-1} n_{\bar{a}}^h}$ is the fraction of probes in $\overline{P_a}$ found in bin b_j.

To compare the signal difference of the tail of the probability distribution, we set $r_{low} = \overline{\mu_a}$, the mean signal intensity of the probes in $\overline{P_a}$, and r_{high} = maximum signal intensity. We set the default number of bins, $c = 20$.

To further stabilize and increase the sensitivity of the Kullback-Leibler divergence on the tail of the probability distribution, two modifications are made. First, we introduce the Anderson-Darling type weight function to the Kullback-Leibler divergence. This gives more weight to the tails than the middle of the distribution. Next, we apply the statistic over the two corresponding cumulative distribution functions instead of their probability density functions. We call our improved Kullback-Leibler divergence the Weighted Kullback-Leibler divergence (*WKL score*):

$$WKL(P_a \mid \overline{\mathrm{P_a}}) = \sum_{j=0}^{k-1} \frac{Q_a(j) \log(\frac{Q_a(j)}{Q_{\bar{a}}(j)})}{\sqrt{Q_{\bar{a}}(j)\left[1 - Q_{\bar{a}}(j)\right]}}$$

where $Q_a(j)$ is the cumulative distribution function of the signal intensities of the probes in P_a found in bin b_j ; $Q_{\bar{a}}(j)$ is the cumulative distribution function of the signal intensities of the probes in $\overline{P_a}$ found in bin b_j.

Thus for each hybridized sample, we compute the *WKL* score of every virus $v_a \in V$. Next, we claim that the distribution of WKL scores of all viruses $v_a \in V$ is approximately normal if there is no virus present in a sample. We empirically verify if our claim is correct by a bootstrapping process: Let n be the number of viruses in V. For each virus $v_k \in V$ where $k = 1, \ldots, n$, we choose $|v_k|$ probe signal intensities from a real dataset D randomly with replacement to form a "perturbed" signal intensity distribution of v_k. Such distribution can mimic the situation where virus v_k is not present in the sample D. Thereafter, *n WKL* scores are generated for the set of n viruses. Next, we check if the n WKL scores follow a normal distribution by the Anderson-Darling test for normality at 95% confidence interval. The bootstrap is repeated 100,000 times. The distribution is found to be normal in more than 99% of the time. (NB: since there are 35 viral genomes represented on our microarray, n =35)

Based on the above discussion, we can test if a sample contains virus(es) by making the following null and alternative hypothesis:

H_0: The distribution of *WKL* scores is normal, i.e. viruses are not present in the sample.

H_1: The distribution of *WKL* scores is not normal, i.e. at least 1 virus is present in the sample.

We proceed to apply the Anderson-Darling test for normality on the distribution of *WKL* scores to reject H_0 with 95% confidence interval. If the distribution of *WKL* scores is not normal, then we exclude the virus with the outlying *WKL* score and apply the Anderson-Darling test again. This process is repeated (to identify the presence of co-infecting pathogens) until H_0 is accepted. We denote the distribution of *WKL* score when H_0 is accepted as the background *WKL* distribution. The viruses excluded are thus very likely to be present in the sample since their *WKL* score does not follow the background *WKL* distribution. In our experiments, we observed that P, the probability that a non-normal distribution occurring by random chance with a given WKL score, in samples which contain a virus is very low ie $P < 1.0 \times 10^{-6}$(obtained via Z-score transformation of WKL score). Box 1 shows the pseudo-code for our virus-detection algorithm.

Given a pathogen microarray data D with virus set V and probe set P,

Let $V_{\text{present}} = \Phi$

Let D_{WKL} be the set of $WKL(P_v \| P_{\bar{v}})$ for all $v \in V$;

1. Determine normality of D_{WKL} with Anderson Darling test for normality. If D_{WKL} is a normal distribution with significance level 0.05, return V_{present}. Else, go to step 2.
2. Find the virus v_a with the highest $WKL(P_a \| P_{a'})$ from D_{WKL}.
   Let $V_{\text{present}} = V_{\text{present}} \cup \{ v_a \}$; $D_{WKL} = D_{WKL} - \{ WKL(P_a \| P_{a'}) \}$; Go to step 1.
3. Remove detected r-signature and verify that WKL distribution is normal.
4. If distribution is not normal, go back to step 2 to find co-infecting pathogen.

Box 1: *Analysis framework of pathogen detection chip data*

<u>References</u>

Stephens, M. A. (1974). *EDF Statistics for Goodness of Fit and Some Comparisons*, Journal of the American Statistical Association, Vol. 69, pp. 730-737

Z-score reference: http://www.animatedsoftware.com/statglos/sgzscore.htm
