# Supplementary material for: Optimization and clinical validation of a pathogen detection microarray
Source: Genome Biol. 2007 May 28;8(5):R93. doi: 10.1186/gb-2007-8-5-r93 (PMC1929155; doi:10.1186/gb-2007-8-5-r93)
Supplement: Additional data file 1 — All files are available for download in PDF, JPG, GIF, TIFF, HTML or ZIP formats as indicated on the webpage [25]. Supplementary methods: sample amplification and microarray protocols (PDF); RT-PCR modeling and amplification efficiency score (AES); pathogen detection algorithm (PDA). Supplementary figures. Figure S1: Probe design schema. Probes (40-mers) were tiled at an average 8-base resolution across each of the 35 viral genomes in the manner depicted above. Numbers represent the start and end positions of each probe. Figure S2: Choice of primer tag in random RT-PCR has significant effect on PCR efficiency. Heatmap of probe signal intensities for a clinical hMPV sample following random RT-PCR using original primer (a) A1 or (b) AES-optimized primer A2. Figure S3: Comparison of amplification efficiency of original primer A1 and AES-optimized primer A2. RNA from patients infected with RSV B (n = 5) or hMPV (n = 3) were reverse-transcribed and amplified using primer A1 or A2 and the percentage of r-signature probes with signal above detection threshold was determined. Figure S4: Diagnostic PCR results for RSV patient 412 show that the patient does not have a coronavirus infection. (a) PCR using pancoronavirus primers. Lane 1, 1 kb ladder; lane 2, blank; lane 3, OC43 coronavirus positive control; lane 4, 229E coronavirus positive control; lane 5, RSV patient 412; lane 6, PCR primers and reagents only, as a negative control. (b) PCR using OC43 specific primers. Lane 1, 50 bp ladder; lane 2, blank; lane 3, OC43 coronavirus positive control; lane 4, RSV patient 412; lane 5, purified RSV from ATCC; lane 6, PCR negative control. (c) PCR using 229E specific primers. Lane 1, 229E coronavirus positive control; lane 2, RSV patient 412; lane 3, PCR negative control; lane 4, 1 kb ladder. Supplementary tables. Table S1: List of genomes represented on the pathogen detection microarray. Table S2: Comparison of E-Predict and PDA algorithms. Pathogen microarray data: data have been [file gb-2007-8-5-r93-S1.zip › Documents and Settings/wongc/Desktop/AES.pdf]

Modeling of RT-PCR for amplification efficiency

The 26-mer primer used for reverse transcription (RT) comprises a fixed 17-mer tag with a 9-mer random tail: 5'-GTT TCC CAG TCA CGA TAN NN NNN NN-3'. In theory, the 9 random oligonucleotides in the tail should bind indiscriminately to all nucleic acids in the patient sample, initiating first strand synthesis. After second strand synthesis, all reversed-transcribed sequences will have 17-mer tags at both ends. These sequences are amplified by PCR, using the 17-mer tag as the primer to generate PCR products where majority are between 500-1000 bp in length.

In our model, v_a represents the pathogen in the clinical sample. To generate a 500-1000 bp PCR product in any region of the genome, defined by positions i and j of v_a requires a primer binding to position i, a reverse primer binding to position j in the anti-sense direction such that $500 \le |i-j| \le 1000$. The binding affinity of a primer is determined by two factors: (1) primer dimer formation and (2) hybridization affinity of the primer to the virus v_a. Genomic regions which can be successfully amplified by virtue of having ideal primer binding locations within 1000 nucleotides can be predicted for by calculating an Amplification Efficiency Score (*AES*) for every position of v_a:

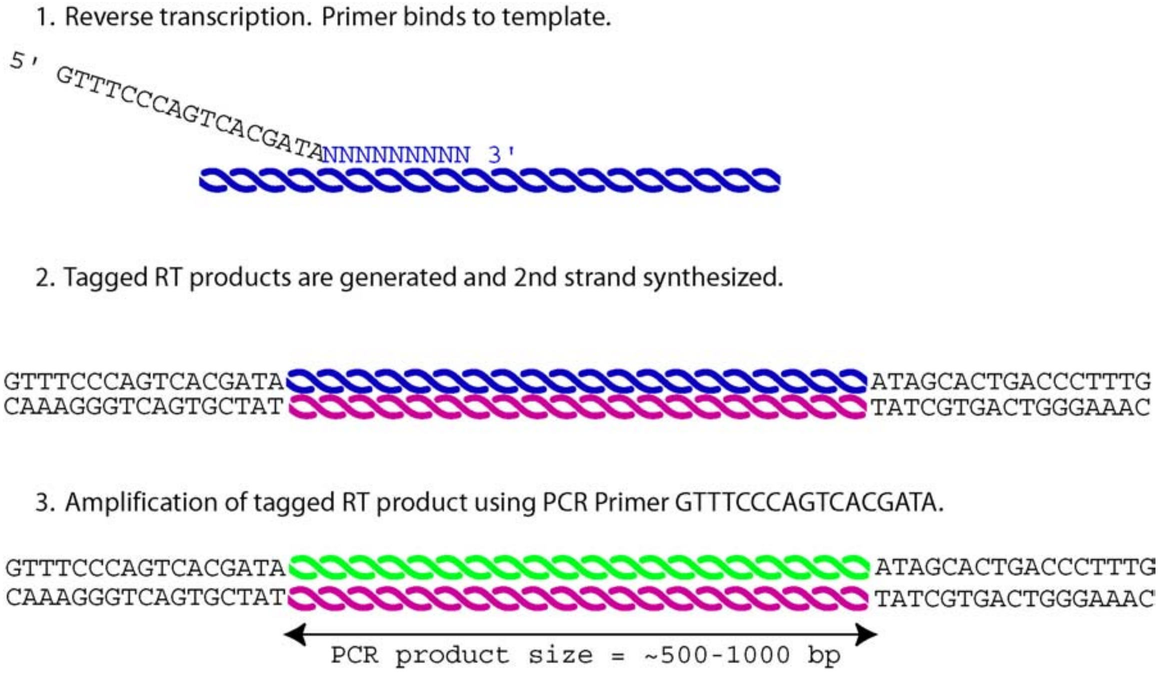

Figure 1: *RT-PCR binding process of random primers on a virus sequence followed by PCR.*

Amplification Efficiency Score (AES)

For each position i of v_a, let $P^f(i)$ and $P^r(i)$ be the probability that a random primer r_i can bind to position i of v_a as forward primer and reverse primer respectively. For simplicity, we assume that a random primer can only bind to v_a if the last 9 nucleotides of the random primer is a substring of the reverse complement of v_a (forward primer) or a substring of v_a (reverse primer; Figure 1). Based on well-established primer design criteria[1], we estimate $P^f(i)$ to be low if r_i forms a significant primer-dimer or has extreme melting temperature. On the other hand, if r_i does not form any significant primer-dimer and has optimal melting temperature, then $P^f(i)$ will be high. If the fixed 17-mer tag of the random primer is similar to v_a, it may also aid in the binding and thus result in a higher $P^f(i)$. Similarly, we compute $P^r(i)$.

The binding of the random primer r_i at position i of v_a as a forward primer affects the quality of the RT-PCR product for the 500 to 1000 nucleotides upstream of position i. Similarly, the binding of the random primer r_i at position i of v_a as a reverse primer affects the quality and coverage of the RT-PCR product for the 500 to 1000 nucleotides downstream of position i. Consider a position x of v_a. All effective primer pairs that reside at positions i and j respectively contribute to the quality of the RT-PCR product at x. Note that $i \le x \le j$ and $500 \le i - j \le 1000$ since our RT-PCR product is 500 to 1000 basepairs long. Thus, an Amplification Efficiency Score, AES_x, for every position x of v_a can be computed by considering the combined effect of all primer pairs that amplifies it:

$$AES_x = \sum_{j=x-1000}^{x} \left\{ P^f(j) \times \sum_{k=\max(x+1,\, j+500)}^{j+1000} P^r(k) \right\}$$

The average AES for a probe of length m beginning at position x is thus:

$$Average\ AES = \frac{\sum_{i=x}^{x+m-1} AES_i}{m}$$

AES threshold predictive of successful RT-PCR

The threshold for amplification efficiency scores for probe selection for a virus v_a is determined by the cumulative distribution function of the *AES* values v_a. Let X be the random variable representing the *AES* values of all probes of v_a. Let k be the number of probes in v_a. Then, we denote the probability that the *AES* value is less than or equal to x be $P(X \le x) = c/k$ where c is the number of probes which have *AES* values less than or equal to x. For a probe p_i at position i of v_a, let x_i be its corresponding *AES* value. Since the signal intensity of a probe is highly correlated to its *AES* value, we estimate $P(p_i \mid v_a)$, the probability that p_i has high signal intensity in the presence of v_a, to be $P(X \le x_i)$. Thus,

$$P(p_i \mid v_a) \approx P(X \le x_i)$$

$$= \frac{c_i}{k}$$

where c_i is the number of probes whose *AES* values are less than or equal to x_i.

For probe selection, probe p_i is selected if $P(p_i \mid v_a) > \lambda$.

To determine the optimal value of λ, we analyzed 4 patient samples hybridized independently to determine the relationship between empirical probe signal intensities and computed AES values. We ranked all probes according to their AES values and binned them into probes in top 10%, top 20%....top 90%, and analyzed the percentage of probes within each bin which had could be detected (Figure 2).

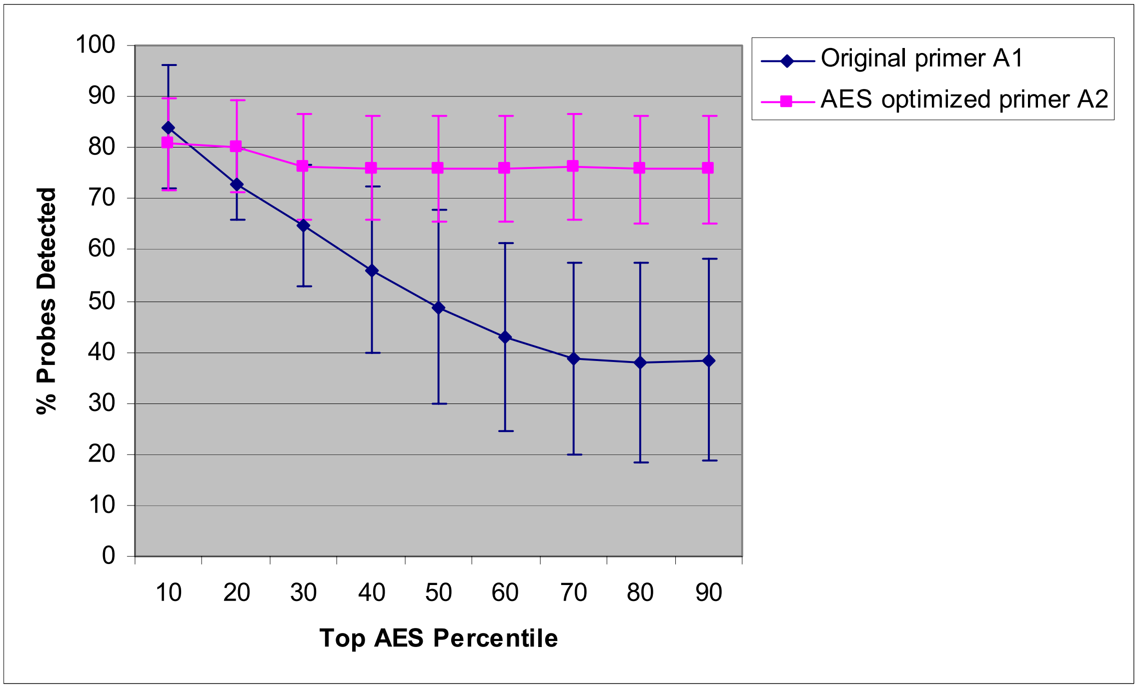

Figure 2. Probe detection is correlated to its AES value.

We observed that >70% of probes with top 20% AES could be reproducibly detected in 4 independent patient samples using the original primer A1. While using probes with higher AES (eg. top 10% AES) would improve reproducibility, this would reduce the number of unique probes remaining for some genomes to <10 at the species level after filters for GC content, melting temperature and cross-hybridization to the human genome, consequently eroding the ability of the array to specifically identify pathogen. Although using an AES-optimized primer A2 resulted in >70% probe detection regardless of AES score, we observed a performance increase to >80% probe detection for those probes with the top 20% AES values.
Thus we selected top 20% AES as a probe design criteria, corresponding to λ = 0.8.

Optimization of PCR primer tag using AES

To design a primer tag which would amplify all 35 genomes represented on the microarray with high efficiency, we generated 10,000 random 17-mer sequences. Those which would form self-dimers were filtered out and AES for every single probe on the microarray was computed for every candidate 17-mer primer tag (using PrimerSelect.java – see software Readme for more information). The primer tag which produced the highest average AES for all 35 viral genomes on the array was selected as the "AES-optimized" primer, A2. Subsequently, we generated a new set of pathogen r-signatures to analyze specimens which were amplified using primer A2. Specimens amplified with original primer A1 were analyzed using r-signatures based on primer A1.

References:

1. Sung WK, Lee WH: **Fast and Accurate Probe Selection Algorithm for Large Genomes.** In: *IEEE Computational Systems Bioinformatics Conference*; 2003; Stanford University, Stanford, CA.
